# Lytic Cell Death in Specific Microglial Subsets Is Required for Preventing Atypical Behavior in Mice

**DOI:** 10.1523/ENEURO.0342-20.2020

**Published:** 2021-01-12

**Authors:** Hsiu-Chun Chuang, Eva K. Nichols, Isabella Rauch, Wei-Cheng Chang, Patrick M. Lin, Rhea Misra, Maiko Kitaoka, Russell E. Vance, Kaoru Saijo

**Affiliations:** 1 Division of Immunology and Pathogenesis, Department of Molecular and Cell Biology, University of California, Berkeley, Berkeley, CA 94720-3200; 2 Howard Hughes Medical Institute, University of California, Berkeley, Berkeley, CA 94720-3200; 3 Helen Wills Neuroscience Institute, University of California, Berkeley, Berkeley, CA 94720-3200

**Keywords:** behavior, cell death, microglia

## Abstract

Microglial cells are known to contribute to brain development and behaviors, but the mechanisms behind such functions are not fully understood. Here, we show that mice deficient in inflammasome regulators, including caspase-1 (*Casp1*), NLR family pyrin domain containing 3 (*Nlrp3*), IL-1 receptor (*Il-1r*), and gasdermin D (*Gsdmd*), exhibit behavior abnormalities characterized by hyperactivity and low anxiety levels. Furthermore, we found that expression of *Casp1* in CX3CR1^+^ myeloid cells, which includes microglia, is required for preventing these abnormal behaviors. Through tissue clearing and 3D imaging, we discovered that small numbers of *Cx3cr1*-GFP^+^ fetal microglial cells formed clusters and underwent lytic cell death in the primitive thalamus and striatum between embryonic day (E)12.5 and E14.5. This lytic cell death was diminished in *Casp1*-deficient mice. Further analysis of the microglial clusters showed the presence of Pax6^+^ neural progenitor cells (NPCs); thus, we hypothesized that microglial lytic cell death is important for proper neuronal development. Indeed, increased numbers of neurons were observed in the thalamic subset in adult *Casp1*^−/−^ brains. Finally, injection of drug inhibitors of NLRP3 and CASP1 into wild-type (WT) pregnant mice from E12.5 to E14.5, the period when lytic cell death was detected, was sufficient to induce atypical behaviors in offspring. Taken together, our data suggests that the inflammasome cascade in microglia is important for regulating neuronal development and normal behaviors, and that genetic or pharmacological inhibition of this pathway can induce atypical behaviors in mice.

## Significance Statement

Microglia support brain development, but the underlying mechanisms are not fully understood. Here, we show that mice deficient for inflammasome cascade protein genes, including NLR family pyrin domain containing 3 (*Nlrp3*), caspase-1 (*Casp1*), IL-1 receptor (*Il-1r*), and gasdermin D (*Gsdmd*), develop behavior abnormalities characterized by hyperactivity and low anxiety. Lytic cell death occurs downstream of inflammasomes and was observed to appear in microglia in spatiotemporal and *Casp1*-dependent manners. Microglial death may be important for the proper differentiation of neural progenitor cells (NPCs), as indicated by increased neuron numbers in specific regions of the brain in *Casp1*-deficient mice. Importantly, injection of NLRP3 and CASP1 inhibitors into pregnant mothers during this lytic death window resulted in offspring with behavior abnormalities. Overall, the death of discrete microglial subsets may be essential for proper NPC development and normal behaviors.

## Introduction

Normal brain development is a series of complex events that can be affected by either genetic or environmental factors. Disruptions in these events can result in neurodevelopmental disorders, such as attention-deficit/hyperactivity disorder (ADHD), autism, and intellectual disabilities characterized by atypical behaviors. Interestingly, cell death by apoptosis has been shown to be essential for normal brain development by controlling the number of neurons in the brain and by helping to establish functional circuits (Yoshida et al., 1998; Yamaguchi and Miura, 2015; Fricker et al., 2018); however, it has not yet been reported whether other types of cell death (lytic cell death such as pyroptosis) are also important for regulating these developmental processes.

Microglial cells are resident innate immune cells in the central nervous system that protect the brain from infection and injury; however, recent studies indicate that they are also crucial for brain development and normal functions (Saijo and Glass, 2011; Ransohoff and El Khoury, 2015; Wolf et al., 2017). For example, a patient with a homozygous mutation in the *CSF1R* gene, which plays essential roles in microglial development, lacked microglia and showed severe defects in brain development (Oosterhof et al., 2019). It has also been reported that microglia-mediated synaptic regulation plays crucial roles in the establishment and maintenance of neural circuits (Paolicelli et al., 2011; Hong et al., 2016). However, the molecular mechanisms behind how microglia shape these circuits and affect their corresponding behaviors are not well understood.

Inflammasomes are cytosolic sensors for a variety of pathogenic and noxious stimuli (Rathinam and Fitzgerald, 2016). In the brain, previous reports suggest that inflammasomes are differentially expressed in different cell types. For example, NLR family pyrin domain containing 1 (NLRP1) and AIM2 were detected in neurons, while NLRP2 and NLRP3 were found in astrocytes (Mamik and Power, 2017; Heneka et al., 2018; Voet et al., 2019). Microglia were also observed to express various inflammasomes, particularly NLRP1, NLRP3, and NLRC4 were highly expressed in this cell type (Walsh et al., 2014; Mamik and Power, 2017; Heneka et al., 2018; Voet et al., 2019). Activated inflammasomes serve as platforms that initiate the cleavage of pro-caspase-1 (CASP1) to CASP1, which then cleaves pro-IL-1β to generate a mature cytokine. CASP1 also cleaves gasdermin D (GSDMD), which is required for subsequent IL-1β secretion, lytic cell death (pyroptosis), and inflammation (Kayagaki et al., 2015; Shi et al., 2015; Extended Data Fig. 1-1*A*).

**Figure 1.**
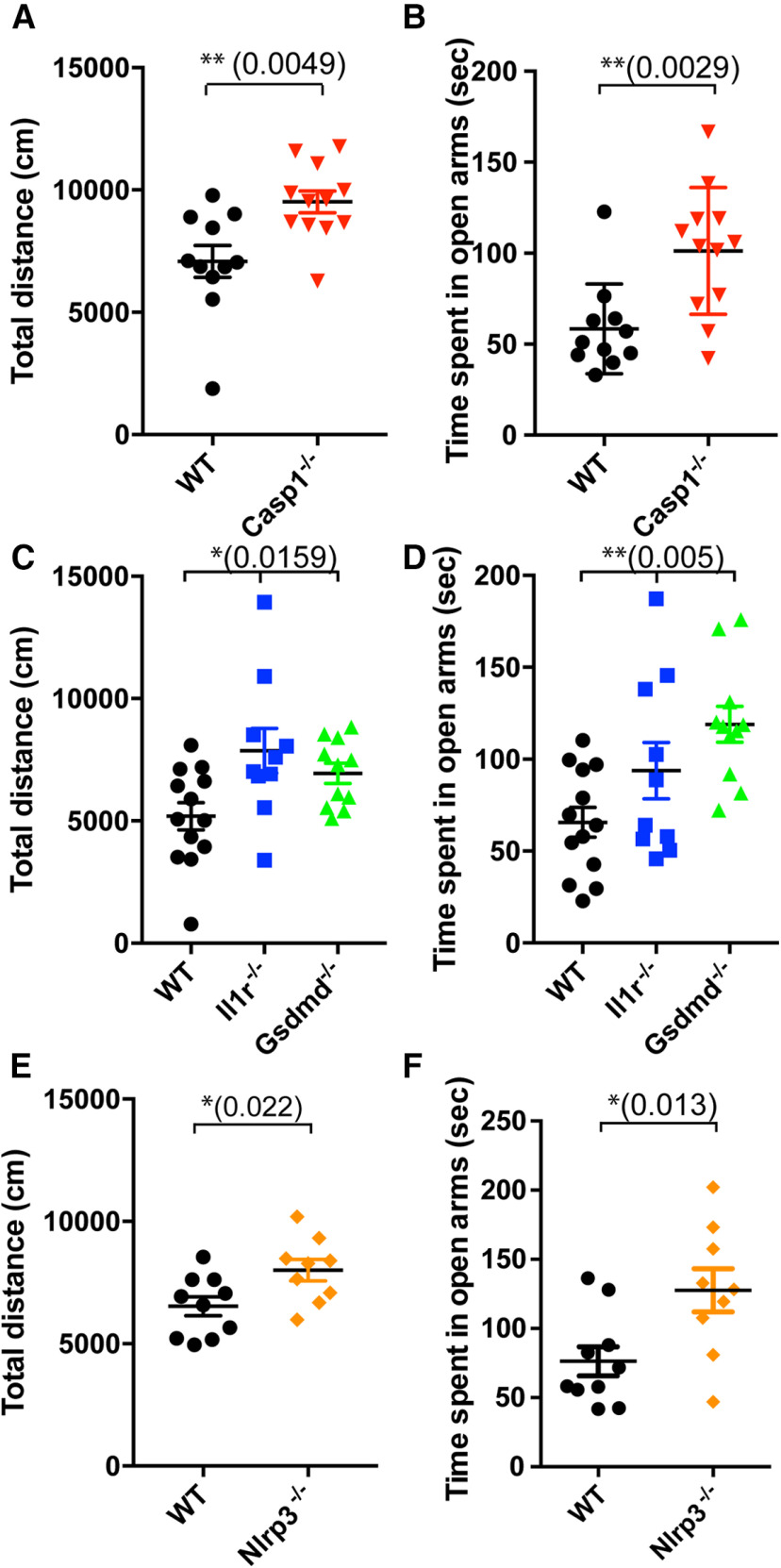
Mice deficient for inflammasome pathway genes exhibit behavior abnormalities. This figure is supported by Extended Data Figure 1-1. ***A***, General activity of male WT (black circles, *N* = 11, from 5 litters) and *Casp1*^−/−^ (red triangles, *N* = 12, from 5 litters) mice was determined by open field assay and is shown as total distance moved (centimeters); *p *=* *0.0049, df = 21, *t *=* *3.142. ***B***, Anxiety levels of male WT and *Casp1*^−/−^ mice were determined using the elevated plus maze assay and are shown as time spent in open arms (seconds); *p *=* *0.0029, df = 21, *t *=* *3.371. ***C***, ***D***, General movement (***C***) and anxiety levels (***D***) of male WT (black circles, *N* = 13, from 4 litters), *Il-1r*^−/−^ (blue squares, *N* = 10, from 3 litters), and *Gsdmd*^−/−^ (green triangles, *N* = 11, from 4 litters) mice were determined as in ***A***, ***B***, respectively. ***C***, *p *=* *0.0159, df = 33, *F* = 4.748. Tukey’s multiple comparison test: WT versus *Il-1r*^−/−^ *p *=* *0.0147, WT versus *Gsdmd*^−/−^ *p *=* *0.1270, *Il-1r*^−/−^ versus *Gsdmd*^−/−^ *p *=* *0.5866. ***D***, *p *=* *0.0050, df = 33, *F* = 6.306. Tukey’s multiple comparison test: WT versus *Il-1r*^−/−^ *p *=* *0.1809, WT versus *Gsdmd*^−/−^ *p *=* *0.0036, *Il-1r*^−/−^ versus *Gsdmd*^−/−^ *p *=* *0.2727. ***E***, ***F***, General movement (***E***) and anxiety (***F***) of male WT (black circles, *N* = 10, from 3 litters) and *Nlrp3*−/− mice (orange diamonds, *N* = 9, from 2 litters) were determined as in ***A***, ***B***, respectively. ***E***, *p *=* *0.0220, df = 17, *t *=* *2.520. ***F***, *p *=* *0.0130, df = 17, *t *=* *2.776. Data shown are individual mice and error bars indicate standard error of the mean (SEM); *p <* *0.05, **p <* *0.01.

10.1523/ENEURO.0342-20.2020.f1-1 Extended Data Figure 1-1 Inflammasome cascade and attention behavior of *Casp1*^−/−^ mice. ***A***, Diagram of the inflammasome cascade. NLRP3, an inflammasome protein, is activated and provides a platform to cleave pro-CASP1 to generate CASP1. CASP1 cleaves pro-GSDMD to GSDMD, which assembles and generates pores in the plasma membrane and induces lytic cell death. CASP1 also cleaves pro-IL-1β to generate mature IL-1β cytokine. ***B***, ***G***, ***I***, Elevated plus maze assay results from the indicated male mice to support Figure 1*B*,*D*,*F* are shown as the time spent in open arms compared to the total time spent on the apparatus (%). ***C***, ***H***, ***J***, Elevated plus maze assay results from the indicated male mice to support Figure 1*B*,*D*,*F* are shown as the number of entries into open arms compared to the total number of entries (%). ***D***, Attention behavior of male WT (*N* = 7, black circles, from 3 litters) and *Casp1*^−/−^ (*N* = 10, red triangles, from 4 litters) mice was determined by the 5-CSRTT and is shown as the accuracy of their responses (%). ***E***, General activity of female WT (black circles, *N* = 4, from 2 litters) and *Casp1*^−/−^ (red squares, *N* = 3, from 2 litters) mice was determined by open field assay and is shown as total distance moved (centimeters). ***F***, Anxiety levels of female WT (black circles, *N* = 5) and *Casp1*^−/−^ (red squares, *N* = 4) mice were determined using the elevated plus maze assay and are shown as the time spent in open arms (seconds). Dots indicate individual animals and error bars show SEM. ***B***, *p *=* *0.0029, df = 21, *t *=* *3.371. ***C***, *p *=* *0.0002, df = 21, *t *=* *4.425. ***D***, *p *=* *0.028, df = 15, *t *=* *2.432. ***E***, *p *=* *0.5129, df = 5, *t *=* *0.704. ***F***, *p *=* *0.9829, df = 7, *t *=* *0.022. ***G***, *p *=* *0.0050, df = 33, *F* = 6.304. Tukey’s multiple comparison test: WT versus *Il-1r*^−/−^ *p *=* *0.1809, WT versus *Gsdmd*^−/−^ *p *=* *0.0036, *Il-1r*^−/−^ versus *Gsdmd*^−/−^ *p *=* *0.2729. ***H***, *p *=* *0.1518, df = 33, *F* = 2.005. Tukey’s multiple comparison test: WT versus *Il-1r*^−/−^ *p *=* *0.2964, WT versus *Gsdmd*^−/−^ *p *=* *0.1717, *Il-1r*^−/−^ versus *Gsdmd*^−/−^ *p *=* *0.9610. ***I***, *p *=* *0.0130, df = 17, *t *=* *2.776. ***J***, *p *=* *0.0035, df = 17, *t *=* *3.392; *p <* *0.05, **p < 0.01; n.s., not significant. Download Figure 1-1, TIF file.

The importance of the inflammasome pathway in protecting the host from infection and injury is well established (Broz and Dixit, 2016). In addition to being a sentinel, it has also been reported that the inflammasome protein NLRP3 in microglia recognizes amyloid-β peptide and induces an inflammatory response that contributes to the pathogenesis of Alzheimer’s disease (Heneka et al., 2013). The idea that NLRP3 plays a role in Alzheimer’s disease has been further supported by recent evidence indicating that NLRP3 regulates tau phosphorylation and aggregation (Ising et al., 2019). Thus, inflammasomes can be involved in the onset and progression of neurologic disorders; however, their role in early brain development is not understood or expected.

Previous literature has shown that mice deficient for interleukin-1 receptor (*Il-1r*) exhibit hyperactivity and low anxiety levels (Murray et al., 2013). Since mature IL-1β is the product of inflammasome activation, we hypothesized that this pathway might be required for normal brain development and behavior. To test our hypothesis, we ran a panel of behavior assays (Table 1) using mice deficient for factors of the inflammasome cascade and found that they exhibited specific behavior abnormalities. Moreover, during normal development, we observed that lytic cell death occurred in subsets of *Cx3cr1*-GFP^+^ brain parenchymal microglial cells, particularly those located in the primitive thalamus and striatum, but this death was notably absent in mice lacking *Casp1*. Furthermore, administration of inhibitors of NLRP3 and CASP1 to wild-type (WT) pregnant female mice at specific gestational days induced behavior abnormalities in their offspring that were similar to those observed in the knock-out mice. Overall, our data suggests that the inflammasome cascade mediated by *Nlrp3*, *Casp1*, *Il-1r*, and *Gsdmd* in microglia is important for normal brain development and for preventing abnormal behaviors in mice.

**Table 1:** Behavior assays

Behavior	Assay
Hyperactivity	Open field assay (total distance they moved)
Anxiety	Elevated plus maze assay
Attention	5-CSRTT assay

The behavior assays used in this study are shown.

## Materials and Methods

### Animals

Both male and female mice (aged two to four months old) were used in this study. All animals were maintained in specific pathogen-free conditions under a 12/12 h light/dark cycle (7 A.M. to 7 P.M.) and were given a standard chow diet and water *ad libitum*. WT C57BL/6J mice were purchased from The Jackson Laboratory. *Casp1*^−/−^*Casp11*^−/−^ mice were on the C57BL/6J background and were kindly provided by A. van der Velden and M. Starnbach (van der Velden et al., 2003). *Casp1*^−/−^, *iCasp1*, *Nlrp3*^−/−^, and *Gsdmd*^−/−^ mice on the C57BL/6J background were generously provided by R. E. Vance (Rauch et al., 2017). Tg(*Cx3cr1*-cre)MW126Gsat mice were generated by the Heintz laboratory at The Rockefeller University and were purchased from MMRRC (University of California Davis). We backcrossed the Tg(*Cx3cr1*-cre)MW126Gsat mice to C57BL/6J mice >10 times before crossing with other strains. *Il-1r*^−/−^ and *Cx3cr1^GFP/GFP^* mice were on the C57BL6/J background and were purchased from The Jackson Laboratory. To normalize gut microbiota, all animals were cohoused in mixed-genotype groups of three to five mice per cage on weaning. If cohousing was not done, their bedding was mixed regularly to normalize the microenvironment. All experiments were approved by the Animal Care and Use Committee and were performed under the institutional guidelines.

### Behavior assays

#### Open field assay

For the experiments with *Casp1*^−/−^ mice, both male and female mice were used, as indicated. For the experiments with *Il-1r*^−/−^, *Gsdmd*^−/−^, *Nlrp3*^−/−^, and *iCasp1* (re-expression and their controls) mice, only male mice were used. We also used only male offspring from inhibitor-injected mothers for this assay. Mice were individually placed in the center of a plastic box (22 × 42.5 × 21 cm) for 1 h and were allowed to freely explore the arena. The data from the first 30 min of exploration were used in our analysis (Komada et al., 2008; Jiang et al., 2010; Chung et al., 2011). Animal movement was monitored by computerized photobeam using the MotorMonitor SmartFrame System (Kinder Scientific).

#### Elevated plus maze assay

We used both male and female *Casp1*^−/−^ mice (as indicated), male *Il-1r*^−/−^, *Gsdmd*^−/−^, *Nlrp3*^−/−^, and *iCasp1* (re-expression and their controls) mice, as well as male offspring from inhibitor-injected mothers for this assay. Mice were individually placed in the center of a platform (5 × 5 cm) of a maze that consisted of two open and two closed arms (30 × 5 cm) that were elevated 30 cm from the floor. Mice were allowed to freely explore the maze for 10 min (Lin and Hsueh, 2014; Nakajima et al., 2019; Shieh and Yang, 2019) and the Smart Video Tracking System (Panlab) was used to determine the time spent in the open and closed arms, as well as the center, during the test. The number of entries into each arm was also analyzed.

#### Five-choice serial reaction time task (5-CSRTT) assay

*Casp1*^−/−^ and WT male mice were used for this assay. The procedures used in this task were as previously described with modifications (Sanchez-Roige et al., 2012). A Plexiglas operator chamber (19 × 22 × 24 cm) with five response apertures and a food magazine was automatically controlled by the software (Packwin, Panlab). The procedure consisted of the pretraining, magazine training, and 5-CSRTT training phases. Before the test and then throughout the entire experiment, the mice were food-restricted (1.8–2 g/d). On the first day of food restriction, the mice were introduced to some reward pellets (TestDiet 14-mg sugar pellets) to familiarize them with their taste. During the pretraining phase, mice were habituated to the chamber with five reward pellets in the food magazine and one reward pellet in each of the five apertures being placed for each operant chamber. The magazine light and all five stimulus lights remained illuminated for the duration of the session. The mice were individually placed in the chamber and allowed to freely explore for 10 min. The habituation session was repeated until all the pellets were consumed. On the day of magazine training, the mice were placed in the chamber for a 4-min period of free exploration, followed by all five stimulus lights being switched on throughout the remainder of the session. After a random nose-poke response was made in one of the five apertures, the mouse was given one pellet in the food magazine. Once the mice earned 20 pellets, they commenced the 5-CSRTT training, which graduates through increasingly challenging stages (stages 1–6) on a schedule of progressively decreasing stimulus duration (SD) and increasing intertrial intervals (ITIs). The percentage of accuracy was calculated as [(number of correct trials/(number of correct and incorrect trials)) × 100]. The number of days taken to achieve the criteria for stage 6 (accuracy >75%, correct trials >20) was recorded to indicate the attention performance. After meeting the criteria of stage 6 for at least two consecutive days, mice were moved forward in the testing schedule in which the variable ITI (5, 8.75, and 12.5 s) was randomly presented with an SD of 1.25 s. The percent accuracy was also calculated in this testing.

### PI injection and tissue clearing

A total volume of 100 μl of propidium iodide (PI; 1.0 mg/ml) solution was intravenously injected into pregnant female mice at specific gestational dates. After 10–30 min of incubation, the mice were killed. Intact fetal bodies were recovered, fixed, and subjected to the CUBIC tissue clearing method (Susaki et al., 2014).

### Light sheet fluorescence microscopy (LSFM) and analysis

We used male embryos that resulted from crossing WT or *Casp1*^−/−^ mice with *Cx3cr1*-GFP mice for this assay. LSFM was performed using the Zeiss Lightsheet Z.1 system and 5× objective set (EC Plan-Neofluar 5×/0.16 detection and LSFM 5×/0.1 illumination lens) at 0.36× zoom. CUBIC R2 solution was used as the refractive index-matched solution. Coronal acquisitions captured the entirety of the embryonic forebrain (∼620 Z slices) with voxel sizes approximately *x* = 2.13 μm, *y* = 2.13 μm, and *z* = 6.16 μm. Imaging data were processed and analyzed by Imaris 9.1.0 software (Bitplane).

### Immunofluorescence staining

We used male WT and *Casp1*^−/−^ embryos and adult brains for this assay. Intact tissues were fixed in 4% paraformaldehyde (PFA) in PBS overnight at 4°C and underwent complete sucrose gradient up to 30% wt/vol in PBS. Tissues were next embedded in a 1:1 mix of TissueTek O.C.T. compound with 30% sucrose in PBS and sectioned (40-μm sections) with a cryostat. Slides were dried and permeabilized with 0.1% Triton X-100 in PBS. After blocking, sections were incubated with primary antibodies (see Table 2) followed by secondary antibodies.

**Table 2:** Information on primary antibodies

Name	Vendor	Catalog #	Dilution
NeuN	Millipore Sigma	ABN78	1:300
Parvalbumin	Millipore Sigma	P3088	1:1000
Iba1	Thermo Fisher	PA5-18039	1:250
Pax6	Eurogentec	PRB-278P-100	1:100

The information on primary antibodies used in this study is shown.

### Drug administration to mice

Pregnant C57BL/6 mice were given three intraperitoneal injections of either VX-765 (50 mg/kg/d, caspase-1 inhibitor, InvivoGen), MCC950 (50 mg/kg/d, NLRP3-inflammasome inhibitor, InvivoGen), or saline as vehicle. Drugs were injected once per day starting on embryonic day (E)12.5 until E14.5. Behavioral assays were performed on the male offspring when they were eight weeks old.

### RNA extraction and RT-qPCR

Total RNA was isolated using the DirectZol kit (Zymo Research). RTs were performed using SuperScript III (ThermoFisher) and qPCR was performed using the KAPA SYBR Green Fast qPCR kit by following the manufacturer’s protocols.

Primers used in this assay were as follows: *Hprt*: F 5′-TCAGTCAACGGGGGACATAAA-3′ and R 5′-GGGGCTGTACTGCTTAACCAG-3′; *Casp1*: F 5′-TGGGACCCTCAAGTTTTGCCC-3′ and R 5′-GGCAAGACGTGTACGAGTGGTT-3′; *Nlrp3*: F 5′-CTTTGCTGCGATCAACAGGCG-3′ and R 5′-TCAAGGCTGTCCTCCTGGCATA-3′; *Cd200*: F 5′-TCACTTGCTCTGCGACTGCC-3′ and R 5′-GGGGTCTTTGACCCGGAGGA-3′; and *Celf4*: F 5′-CAAGGAGCGCACAATGCGAC-3′, R 5′-ATGAGGGCTGCTTGCTGCTG-3′.

### Nissl staining and stereology

Three adult age-matched WT and *Casp1*^−/−^ male mice underwent transcardial perfusion with PBS and 4% PFA in PBS and postfixation overnight in 4% PFA in PBS. Brains were embedded in O.C.T. compound (Sakura VWR) before serial sectioning at 40 μm in a cryostat. For Nissl staining, sections were stained in Cresyl violet solution (0.3% wt/vol Cresyl violet acetate in water plus 0.3% glacial acetic acid v/v; Sigma C-1791 and 537020) for 45 min. Sections were rinsed in ddH_2_O and subjected to an ethanol dehydration series (1-min steps; 70%, 95%, 100% EtOH) before clearing in xylenes for 3 min (Sigma 214736). Sections were mounted in Permount and dried for 24–48 h before imaging.

The Cavalieri volume estimation method (MicroBrightField) was used to estimate the volume of the thalamus of three adult age-matched WT and *Casp1*^−/−^ mouse brains (from approximately bregma −1 mm to +4 mm). Nissl-stained sections (40 μm) were visualized using the brightfield setting on a BX51 microscope (Olympus) connected to a computer running Stereo Investigator 11.03 software (MicroBrightField). Afterwards, markers were placed (using a grid spacing of 50 μm and a section evaluation interval of 6) over these regions for a total of 11 slices throughout the thalamus. Contours were drawn according to regions defined by the Allen Brain Atlas. Total volumes were then determined using the built-in software estimator. All volume estimations except for one possessed a Gundersen coefficient of error <0.10.

### Statistical analysis

Data are shown as averages with error bars indicating SEM. Sample sizes were calculated based on the Boston University IACUC spread sheet. Statistical analysis was performed using Prism 7 (GraphPad) software. We used the Student’s *t* test to compare two different groups, and we used the one-way ANOVA with *post hoc* tests (Bartlett’s test and Brown–Forsythe test) to compare three different groups. In addition to one-way ANOVA, Tukey’s multiple comparison tests were performed to show the individual *p* values. The data were provided to the figure legends. The Fisher’s exact test was used to analyze contingency tables to determine the probability that the distributions were not because of chance. Table 3 shows the *t* values, df, and *p* values for the Student’s *t* tests, and Table 4 shows the sum-of-squares (SS), df, the mean squares (MS), F ratios, and *p* values for the one-way ANOVAs between columns, within columns, and total for the indicated figures; *p* < 0.05 was considered to be statistically significant.

**Table 3:** Information for Student’s *t* tests

Figure	*t* value	df	*p* value
Fig. 1*A*	3.142	21	0.0049
Fig. 1*B*	3.371	21	0.0029
Fig. 1*E*	2.520	17	0.0220
Fig. 1*F*	2.776	17	0.0130
Fig. 4*C*	3.694	4	0.0209
Fig. 4*E*	0.336	4	0.7533
Extended Data Fig. 1-1*B*	3.371	21	0.0029
Extended Data Fig. 1-1*C*	4.425	21	0.0002
Extended Data Fig. 1-1*D*	2.432	15	0.0280
Extended Data Fig. 1-1*E*	0.704	5	0.5129
Extended Data Fig. 1-1*F*	0.022	7	0.9829
Extended Data Fig. 1-1*I*	2.776	17	0.0130
Extended Data Fig. 1-1*J*	3.392	17	0.0035
Extended Data Fig. 2-1*A*	688.100	2	<0.0001
Extended Data Fig. 2-1*B*	1319.000	2	<0.0001
Extended Data Fig. 2-1*C*	598.900	2	<0.0001
Extended Data Fig. 2-1*D*	220.900	2	<0.0001
Extended Data Fig. 3-1*D*	3.935	30	0.0005
Extended Data Fig. 4-1, cerebrum	0.191	4	0.8580
Extended Data Fig. 4-1, thalamus	0.691	4	0.5274
Extended Data Fig. 4-1, striatum	0.084	4	0.9372

For the Student’s *t* tests used in our analyses, the *t* values, df, and *p* values are summarized.

**Table 4:** Information for one-way ANOVA

	SS	df	MS	*F*_(dfn,dfd)_	** ***p*
Fig. 1*C*					
Between columns	43308629	2	21654315	*F*_(2,31)_ = 4.748	*p *=* *0.0159
Within columns	141389895	31	4560964		
Total	184698524	33			
Fig. 1*D*					
Between columns	17029	2	8514	*F*_(2,31)_ = 6.306	*p *=* *0.0050
Within columns	41856	31	1350		
Total	58885	33			
Fig. 2*B*					
Between columns	18214401	2	9107200	*F*_(2,22)_ = 3.247	*p *=* *0.0581
Within columns	61708704	22	2804941		
Total	79923105	24			
Fig. 2*C*					
Between columns	21480	2	10740	*F*_(2,26)_ = 15.97	*p *<* *0.0001
Within columns	17489	26	672.7		
Total	38969	28			
Fig. 5*B*					
Between columns	2577676	2	1288838	*F*_(2,26)_ = 3.614	*p *=* *0.0412
Within columns	9272962	26	356652		
Total	11850639	28			
Fig. 5*C*					
Between columns	14905	2	7453	*F*_(2,26)_ = 3.92	*p *=* *0.0325
Within columns	49424	26	1901		
Total	64329	28			
Extended Data Fig. 1-1*G*					
Between columns	473	2	236.5	*F*_(2,31)_ = 6.304	*p *=* *0.0050
Within columns	1163	31	37.51		
Total	1636	33			
Extended Data Fig. 1-1*H*					
Between columns	136.1	2	68.07	*F*_(2,31)_ = 2.005	*p *=* *0.1518
Within columns	1053	31	33.96		
Total	1189	33			
Extended Data Fig. 2-1*E*					
Between columns	11.75	2	5.876	*F*_(2,8)_ = 16.19	*p *=* *0.0015
Within columns	2.904	8	0.363		
Total	14.66	10			
Extended Data Fig. 2-1*F*					
Between columns	597.6	2	298.8	*F*_(2,26)_ = 15.92	*p *<* *0.0001
Within columns	488	26	18.77		
Total	1086	28			
Extended Data Fig. 2-1*G*					
Between columns	230.5	2	115.3	*F*_(2,26)_ = 3.902	*p *=* *0.0330
Within columns	768	26	29.54		
Total	998.5	28			
Extended Data Fig. 5-1*B*					
Between columns	414	2	207	*F*_(2,26)_ = 3.921	*p *=* *0.0325
Within columns	1373	26	52.8		
Total	1787	28			
Extended Data Fig. 5-1*C*					
Between columns	78.67	2	39.34	*F*_(2,26)_ = 1.724	*p *=* *0.1981
Within columns	593.2	26	22.82		
Total	671.9	28			

For our one-way ANOVA analyses, the SS, df, the MS, F ratios, and *p* values used to assess the variation between columns, within columns, and total are summarized.

**Figure 2.**
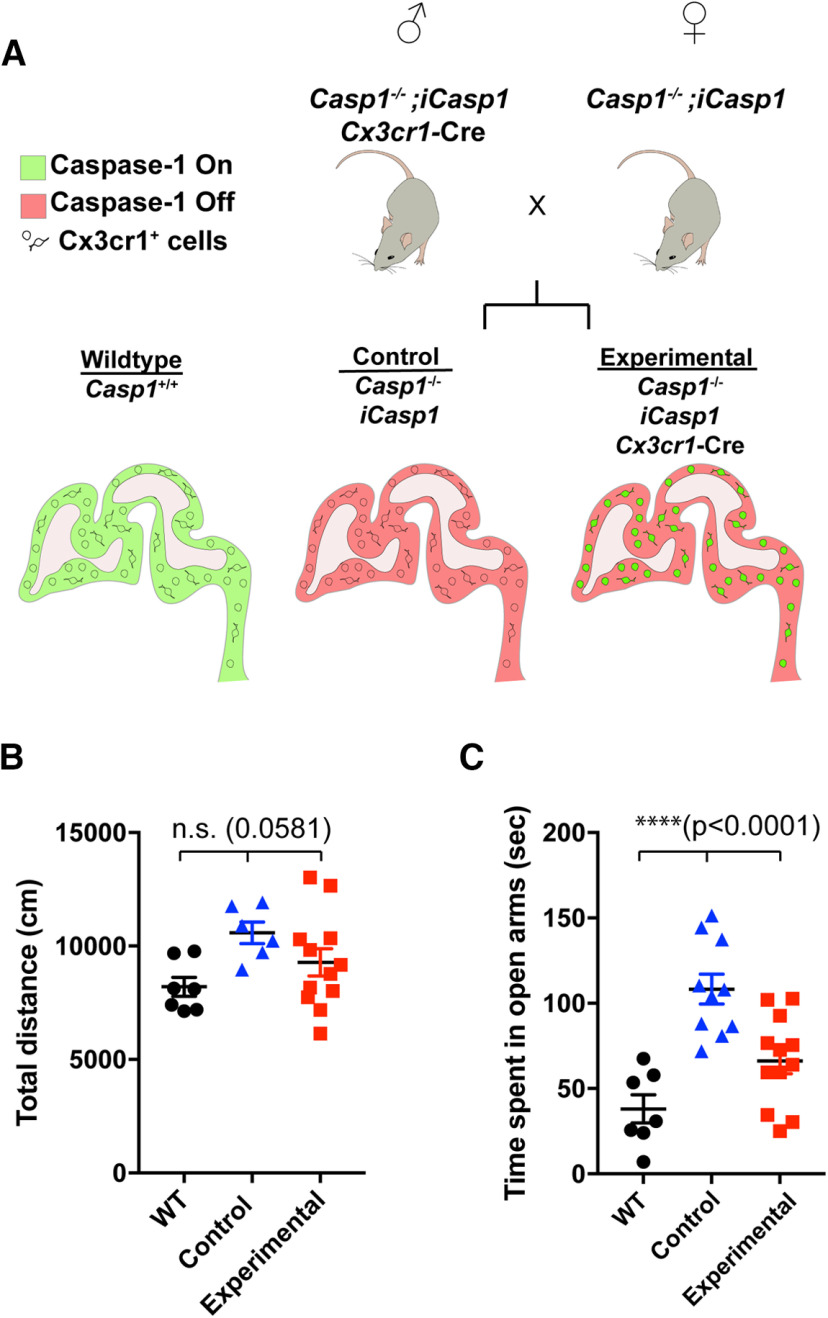
*Casp1* re-expression in CX3CR1^+^ cells restores normal behavior. This figure is supported by Extended Data Figure 2-1. ***A***, Experimental design is shown. Three mouse lines (*Casp1*^−/−^, *iCasp1*, and *Cx3cr1*-Cre) were crossed to generate *Casp1*^−/−^; *iCasp1*; *Cx3cr1*-Cre positive mice (experimental) and *Casp1*^−/−^; *iCasp1*; Cre negative littermates (control). Mice expressing Cre had induced expression of *Casp1* in CX3CR1^+^ cells, but not in CX3CR1^–^ cells. ***B***, General movement of male WT (black circles, *N* = 7, from 3 litters), littermate control (blue triangles, *N* = 6, from 4 litters), and experimental (red squares, *N* = 12, from 8 litters) mice was determined by open field assay and is shown as total distance moved (centimeters); *p *=* *0.0581, df = 24, *F* = 3.247. Tukey’s multiple comparison test: WT versus control *p *=* *0.0997, WT versus experimental *p *=* *0.5502, control versus experimental *p *=* *0.3072. ***C***, Anxiety levels of male WT (black circles, *N* = 7, from 3 litters), control (blue triangles, *N* = 10, from 6 litters), and experimental (red squares, *N* = 12, from 8 litters) mice were determined by elevated plus maze assay and are shown as time spent in open arms (seconds); *p *<* *0.0001, df = 28, *F* = 15.97. Tukey’s multiple comparison test: WT versus control *p *<* *0.0001, WT versus experimental *p *=* *0.0760, control versus experimental *p *=* *0.0023. Data indicates individual animals and error bars are shown as SEM; ****p <* *0.0001; n.s., not significant.

**Figure 3.**
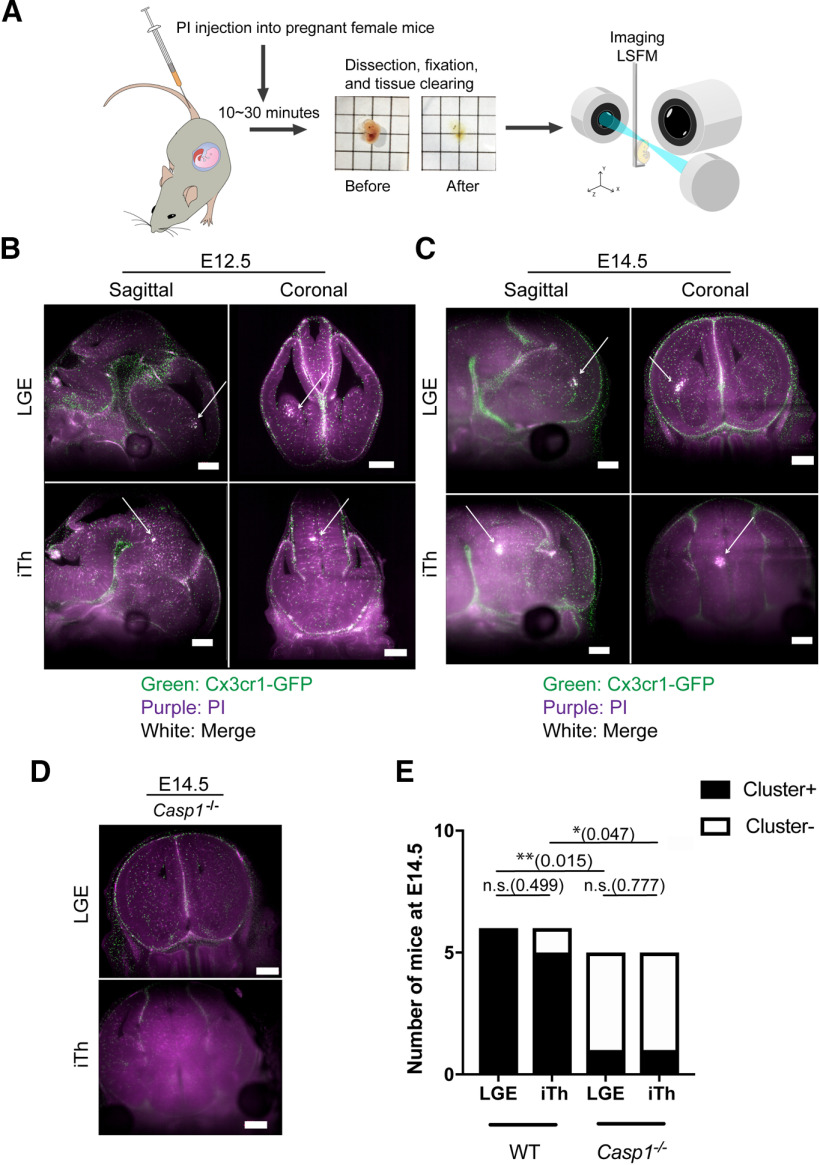
Lytic cell death occurs in a spatiotemporal manner in the fetal brain. This figure is supported by Extended Data Figure 3-1 and Movies 1, 2. ***A***, Experimental scheme is shown. PI was injected into pregnant female mice at specific gestational stages. Ten to 30 min after PI injection, total fetal bodies were recovered, cleared, and imaged by LSFM. ***B***, Representative sagittal and coronal LSFM images of the LGE and primitive iTh of a WT E12.5 fetal brain (male, *N* = 6, from 2 litters) are shown. Arrows indicate the cluster. Green, GFP; purple, PI. Scale bars: 500 μm. ***C***, Representative images of a WT E14.5 fetal brain (male, *N* = 6, from 3 litters) are shown. Arrows indicate the cluster. Green, GFP; purple, PI. Scale bars: 500 μm. ***D***, Representative coronal LSFM images of a *Casp1*^−/−^ brain (male, *N* = 5, from 3 litters) at E14.5 are shown. Scale bars: 500 μm. ***E***, Numbers of mice that exhibited clusters in the LGE and iTh at E14.5 are shown; *p <* *0.05, **p <* *0.01; n.s., not significant.

**Figure 4.**
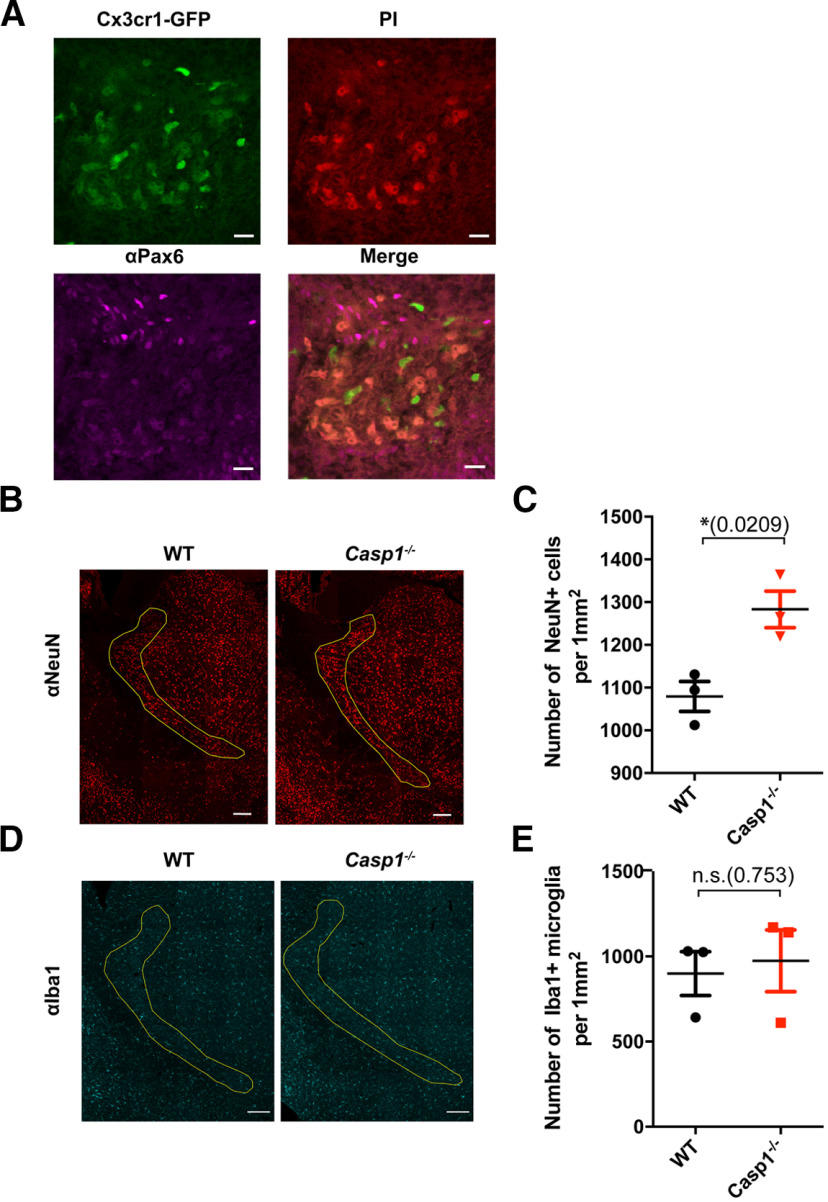
Numbers of neurons but not microglia are increased in the TRN in adult *Casp1*^−/−^ mice. This figure is supported by Extended Data Figure 4-1. ***A***, Enlarged image of the GFP^+^PI^+^ cluster shown in Figure 3. Mice were injected with PI as shown in Figure 3*A*, and fetal brain slices were stained with anti-Pax6 antibody. Images of *Cx3cr1*-GFP (top left), PI (top right), anti-Pax6 (bottom left), and merged signals are shown. Scale bar: 50 μm. ***B***, Representative images of anti-NeuN staining signals from male *Casp1*^−/−^ (*N* = 3, right, from 2 litters) and sex/age-matched WT (*N* = 3, left, from 2 litters) adult brains are shown. TRN regions are outlined in yellow. Scale bar: 200 μm. ***C***, Average anti-NeuN signals from *Casp1*^−/−^ (red triangles) and WT (black circles) mice are shown as cell number/mm^2^. Dots indicate individual animals and are the average cell numbers from a total of six serial sections stained as shown in ***B***; *p *=* *0.0209, df = 4, *t *=* *3.694. Error bars indicate SEM; *p <* *0.05. ***D***, Representative images of anti-Iba1 antibody staining in the TRNs (regions outlined in yellow) of male WT (*N* = 3, left, from 2 litters) and *Casp1*^−/−^ (*N* = 3, right, 2 litters) mice are shown. Scale bar: 200 μm. ***E***, Average number of Iba1^+^ cells in *Casp1*^−/−^ (red triangles) and WT (black circles) mice are shown as mean cell number/mm^2^. Dots indicate individual animals and are the average of cell numbers from a total of six serial sections stained as shown in ***D***; *p *=* *0.7533, df = 4, *t *=* *0.336. Error bars indicate SEM; n.s., not significant.

**Figure 5.**
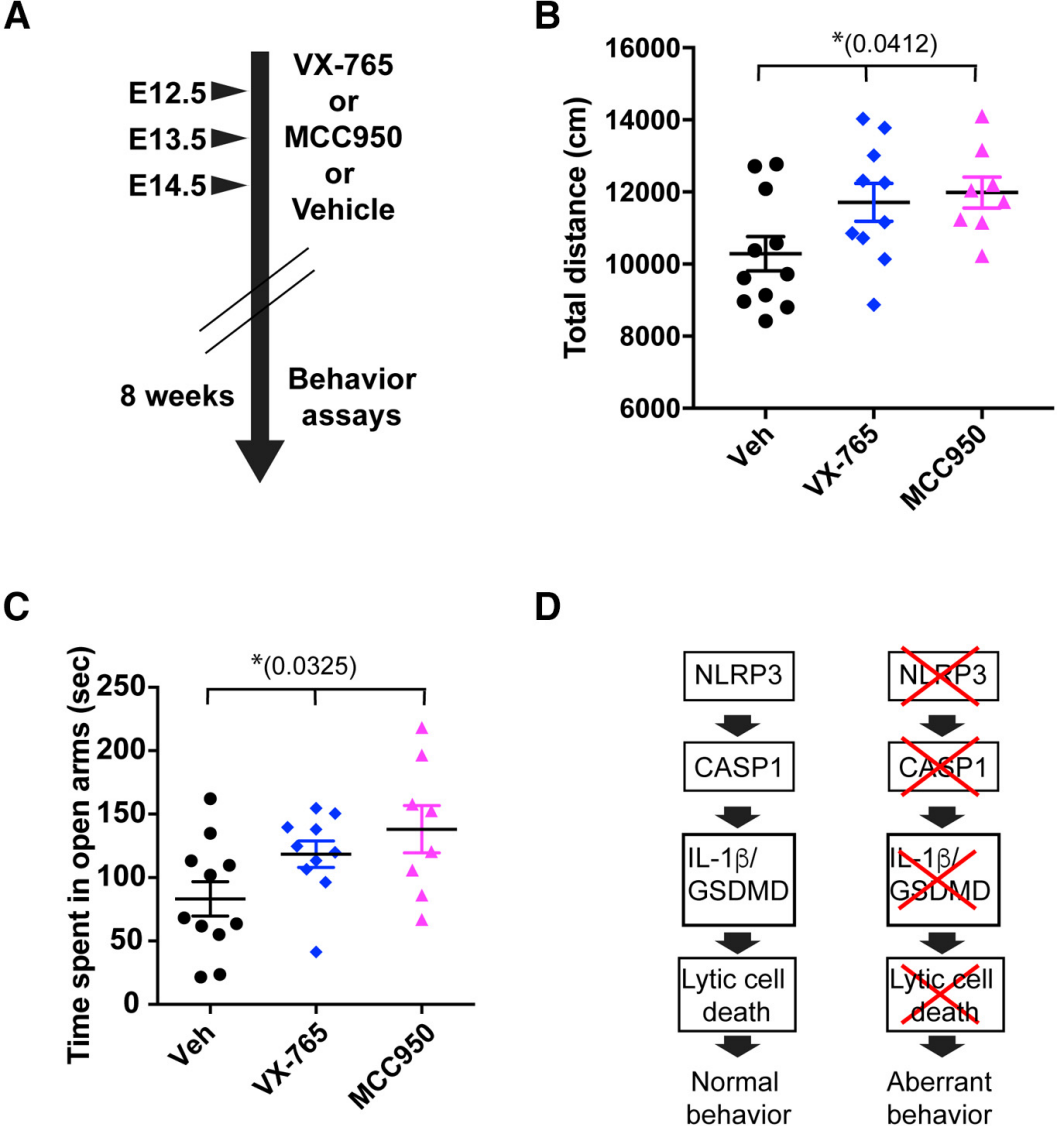
Fetal exposure to inflammasome inhibitors leads to aberrant behaviors. This figure is supported by Extended Data Figure 5-1. ***A***, Experimental scheme is shown. Pregnant WT female mice were injected with inflammasome inhibitors (VX-765 or MCC950 to inhibit CASP1 or NLRP3, respectively) or control (Veh: vehicle) at E12.5, E13.5, and E14.5. Behaviors of the male offspring of injected mice were tested at eight weeks old. ***B***, General activity of the male offspring of control-injected (vehicle; black circles, *N* = 11, from 3 litters), VX-765-injected (blue diamonds, *N* = 10, from 3 litters), and MCC950-injected mothers (pink triangles, *N* = 8, from 3 litters) was determined by open field assay and is shown as the total distance moved (centimeters); *p *=* *0.0412, df = 28, *F* = 3.614. Tukey’s multiple comparison test: Veh versus VX-765 *p* = 0.0963, Veh versus MCC950 *p *=* *0.0591, VX-765 versus MCC950 *p *=* *0.9250. Dots indicate individual animals, and error bars indicate SEM. ***C***, Anxiety levels in the indicated offspring were determined by elevated plus maze assay and are shown as time spent in open arms (seconds); *p *=* *0.0325, df = 28, *F* = 3.92. Tukey’s multiple comparison test: Veh versus VX-765 *p *=* *0.1736, Veh versus MCC950 *p *=* *0.0307, VX-765 versus MCC950 *p *=* *0.6151. Data are shown as individual animals, and error bars indicate SEM; *p <* *0.05; n.s., not significant. ***D***, Working model is shown. Activation of the NLRP3-CASP1-GSDMD/IL-1β cascade in fetal microglia is required for normal brain development. Genetic or pharmacological disruption of this pathway results in aberrant behaviors.

10.1523/ENEURO.0342-20.2020.f3-1 Extended Data Figure 3-1 Quantification and qualification of clusters in WT and *Casp1* re-expression mice. ***A***, Numbers of WT mice (male, *N* = 6) that exhibited clusters in the LGE and iTh at E12.5 are shown; n.s., not significant. ***B***, Cell numbers in the clusters from the LGE and iTh at E14.5 are shown. ***C***, A representative brightfield image of the cluster including swollen microglial cells. Scale bar: 100 μm. ***D***, Diameters of GFP^+^ and GFP^+^PI^+^ cells in the clusters are shown; *p *=* *0.0005, df = 30, *t *=* *3.935. Diameters of individual cells and the averages are shown. Error bars indicate SEM; ***p <* *0.001. ***E***, *Casp1*^−/−^; *iCasp1*; *Cx3cr1*-Cre mice express GFP upon Cre-mediated recombination. Mice were injected with PI as shown in Figure 3A. Representative brightfield (top left), *Cx3cr1*-GFP staining signal (top right), PI signal (bottom left), and merged (bottom right) images are shown. Scale bar in the bright field image indicates 230 μm and the others indicate 130 μm. Download Figure 3-1, TIF file.

## Results

### Mice deficient for genes regulating the inflammasome Cascade exhibit behavior abnormalities

Since a previous report showed that *Il-1r*^−/−^ mice exhibit aberrant behaviors, such as hyperactivity and low anxiety (Murray et al., 2013), we decided to use a panel of behavior assays to assess the global impact of inflammasomes on mouse brain development (Table 1). We first investigated the role of CASP1 in mouse brain because it is a common downstream target of inflammasomes and because multiple inflammasome complexes are expressed in microglia and macrophages (Walsh et al., 2014). We found that male *Casp1*^−/−^ mice showed hyperactivity (Fig. 1*A*) and low anxiety levels (Fig. 1*B*; Extended Data Fig. 1-1*B*,*C*), as determined by the open field and elevated plus maze assays, respectively, that were similar to the behavior abnormalities observed in *Il-1r*^−/−^ mice. We also noticed that male *Casp1*^−/−^ mice showed less attention to light stimuli compared with WT mice, as measured by the 5-CSRTT (Extended Data Fig. 1-1*D*; Sanchez-Roige et al., 2012). However, we did not observe other behavioral abnormalities, including decreased sociability, defective learning and memory, or increased restricted repetitive behavior (data not shown). Interestingly, these behavior changes were only observed in male, but not female, *Casp1*^−/−^ mice [Fig. 1*A*,*B* (male); Extended Data Fig. 1-1*E*,*F* (female)], which is consistent with several reports suggesting that some neurodevelopmental disorders show sex dimorphisms (Rucklidge, 2010).

Since both GSDMD and pro-IL-1β are cleaved by CASP1 (Extended Data Fig. 1-1*A*) as part of the inflammasome pathway, we also used *Gsdmd-*deficient and *Il-1r*-deficient mice (Kayagaki et al., 2015; Shi et al., 2015) in our behavior studies. Our results showed that *Gsdmd*^−/−^ mice exhibited hyperactivity (Fig. 1*C*) and low anxiety (Fig. 1D; Extended Data Fig. 1-1*G*,*H*). Furthermore, consistent with the previous report, we observed behavior abnormalities in *Il-1r*^−/−^ mice, although to a lesser extent (Fig. 1*C*,*D*; Extended Data Fig. 1-1*G*,*H*). Next, we hypothesized that NLRP3 might be the inflammasome protein responsible for the activation of CASP1 in the fetal brain because NLRP3 detects tissue damage rather than infection (Cassel and Sutterwala, 2010) and because the developing brain in the uterus should be a sterile environment. In addition, NLRP3 could be activated by ATP and ions released from dying/dead cells, given the widespread death of newly differentiated neurons during fetal brain development (Watson et al., 2012). To test this hypothesis, we ran our behavior assays on *Nlrp3*^−/−^ mice and found that they also exhibited hyperactivity (Fig. 1*E*) and low anxiety (Fig. 1*F*; Extended Data Fig. 1-1*I*,*J*). From these results, we concluded that the NLRP3-CASP1-GSDMD/IL-1β pathway is required for establishing normal behavior in mice.

### Re-expression of *Casp1* in CX3CR1^+^ cells restores normal behaviors

The expression of chemokine receptor CX3CR1 is characteristic for myeloid-lineage cells, including microglial cells. In the brain, CX3CR1^+^ cells in the parenchyma are considered microglial cells; however, there are some neutrophils and subsets of macrophages in the meninges and blood vessels that also express CX3CR1 (Mizutani et al., 2012; Wolf et al., 2013). Our gene expression studies showed that *Casp1* and *Nlrp3* mRNAs were highly expressed in CX3CR1^+^ cells, including microglia and macrophages, isolated from fetal brain (Extended Data Fig. 2-1*A*,*B*). In contrast, CX3CR1^–^ cells displayed high expression levels of *Cd200* and *Celf4* mRNAs, which are associated with neural cells (Extended Data Fig. 2-1*C*,*D*; Haimon et al., 2018). Because the *Casp1*^−/−^ mice used in our initial studies were conventional knock-out mice in which *Casp1* was deleted from all cell types, *Casp1*^−/−^ mice expressing a Cre-inducible *Casp1* allele (*Rosa26-LoxP-STOP-LoxP-Casp1-IRES-GFP*, *iCasp1* mice; Rauch et al., 2017) were crossed to BAC transgenic mice expressing Cre under the control of the *Cx3cr1* promoter, a commonly used Cre-driver enriched in microglia (Wolf et al., 2013), so that *Casp1* would be re-expressed in CX3CR1^+^ cells. The resulting *Casp1*^−/−^; *iCasp1*; *Cx3cr1*-Cre (experimental) mice and their *Casp1*^−/−^; *iCasp1* littermate controls (control, equivalent to *Casp1*^−/−^; Fig. 2*A*) as well as WT mice were then used in our previously described behavior assays (Table 1) to verify that re-expression of *Casp1* in immune cells is necessary for preventing behavior abnormalities and ensuring normal brain development. Remarkably, re-expression of *Casp1* in CX3CR1^+^ cells (Extended Data Fig. 2-1*E*) resulted in animals without hyperactivity (Fig. 2*B*) or low anxiety levels (Fig. 2*C*; Extended Data Fig. 2-1*F*,*G*); thus, we concluded that *Casp1* expression in CX3CR1^+^ myeloid cells, including microglial cells, plays an essential role in establishing normal behaviors.

10.1523/ENEURO.0342-20.2020.f2-1 Extended Data Figure 2-1 *Casp1* is expressed in microglia from fetal brains and adult *Casp1*^−/−^; *iCasp1*; *Cx3cr1*-Cre mice. ***A–D***, mRNA expressions of *Casp1* (***A***), *Nlrp3* (***B***), *Cd200* (***C***), and *Celf4* (***D***) in *Cx3cr1*-GFP^+^ and *Cx3cr1*-GFP^-^ cells from male fetal brain at E14.5, as determined by RT-qPCR. ***A***, *p *<* *0.0001, df = 2, *t *=* *688.100. ***B***, *p *<* *0.0001, df = 2, *t *=* *1319.000. ***C***, *p *<* *0.0001, df = 2, *t *=* *598.900. ***D***, *p *<* *0.0001, df = 2, *t *=* *220.900. Data are shown as the average of duplicates, and error bars indicate SEM. ***E***, Microglial cells were isolated from the brains of adult male WT (black circles), control (blue rectangles), and experimental (red rectangles) mice and the mRNA expressions of *Casp1* were determined by RT-qPCR. ***F***, Elevated plus maze assay results supporting Figure 2*C* are shown as the time spent in open arms compared to the total time spent on the apparatus (%). ***G***, Elevated plus maze assay results supporting Figure 2*C* are shown as the number of entries into open arms compared to the total number of entries (%). ***E***, *p *=* *0.0015, df = 10, *F* = 16.19. Tukey’s multiple comparison test: WT versus control *p *=* *0.2260, WT versus experimental *p *=* *0.0230, control versus experimental *p *=* *0.0013. ***F***, *p *<* *0.0001, df = 28, *F* = 15.92. Tukey’s multiple comparison test: WT versus control *p *<* *0.0001, WT versus experimental *p *=* *0.0759, control versus experimental *p *=* *0.0024. ***G***, *p *=* *0.0330, df = 28, *F* = 3.902. Tukey’s multiple comparison test: WT versus control *p *=* *0.0743, WT versus experimental *p *=* *0.0340, control versus experimental *p *=* *0.9469. Data indicates individual mice and averages are shown. Error bars indicate SEM; **p <* *0.01, ****p <* *0.0001. Download Figure 2-1, TIF file.

### Lytic cell death occurs in a spatiotemporal manner in the mouse fetal brain

Lytic cell death, downstream of the inflammasome pathway, has been described in macrophages and dendritic cells as well as in aged microglia in the context of Alzheimer’s disease (Heneka et al., 2013). However, it is not known whether microglia undergo lytic cell death during fetal brain development. Because mice deficient for *Gsdmd*, a factor that is essential for the induction of lytic cell death (Shi et al., 2015), exhibited behavior abnormalities similar to those observed in *Casp1*-deficient mice (Fig. 1*A–D*), we hypothesized that fetal microglia might undergo lytic cell death. To investigate whether microglial cell death occurs during early brain development, PI (a DNA dye) was used to visualize dying microglia. PI is able to stain these cells because during lytic cell death, plasma membrane integrity is lost, allowing the dye into the cells (Fink and Cookson, 2005; Zhang et al., 2018). For this assay, PI was injected into pregnant WT females that had been crossed with *Cx3cr1^GFP/GFP^* male mice at specific days during fetal brain development. The genotype of the resulting offspring was *Cx3cr1^GFP/+^*, with microglia that were labeled with GFP (Jung et al., 2000). While it is known that *Cx3cr1* is expressed in non-microglial cells outside the brain, *Cx3cr1*-GFP^+^ cells in the brain parenchyma are considered microglia (Mizutani et al., 2012). Following PI injection, embryos were recovered, the tissue was cleared using the CUBIC method (Susaki et al., 2014), and the brains were imaged via LSFM (Fig. 3*A*). Interestingly, our imaging revealed discrete clusters of GFP^+^PI^+^ cells in both the lateral ganglionic eminence (LGE), which matures into a major part of the striatum, and the intermediate thalamus (iTh) starting at E12.5 (Fig. 3*B*; Extended Data Fig. 3-1*A*). These clusters were also noted in both the LGE and iTh at E14.5 (Fig. 3*C*,*E*; Extended Data Fig. 3-1*B*; Movie 1), coincident with active neurogenesis and neuronal cell death (Watson et al., 2012). Clusters were comprised of 20–200 microglial cells, with averages of 107 and 85 cells/cluster in the LGE and iTh, respectively, at E14.5 (Extended Data Fig. 3-1*B*) and fewer cells at E12.5 (data not shown). To determine whether the cells in the clusters experienced lytic cell death, we measured the diameters of GFP^+^ and GFP^+^PI^+^ cells and found that GFP^+^PI^+^ cells had significantly larger diameters, suggesting that these cells had lost membrane integrity and were swollen (Extended Data Fig. 3-1*C*,*D*).

Since CASP1 activation is required for lytic cell death (Extended Data Fig. 1-1*A*), we next tested whether the observed cell death was impaired in *Casp1*^−/−^ mice. Using *Cx3cr1^GFP/GFP^* mice crossed with *Casp1*^−/−^ mice, we found that the formation of GFP^+^PI^+^ cell clusters in the LGE and iTh was significantly reduced in *Casp1*^−/−^; *Cx3cr1*-GFP offspring at E14.5 (Fig. 3*D*,*E*; Movie 2). Conversely, in *Casp1*^−/−^; *iCasp1*; *Cx3cr1*-Cre (experimental) mice, which express GFP following Cre-mediated recombination (Rauch et al., 2017; Fig. 2*A*), we observed GFP^+^ cells that were also positive for PI signal (Extended Data Fig. 3-1*E*). Overall, these results suggest that microglia in the LGE and iTh undergo lytic cell death during fetal brain development at E12.5–E14.5 in a *Casp1*-dependent manner.

### The thalamic reticular nucleus (TRN) region in *Casp1*^−/−^ brains has increased numbers of neurons

In mice, neural progenitor cells (NPCs) start to differentiate at E12.5, coincident with the microglial lytic cell death that we observed in the fetal brain. Therefore, we hypothesized that defective microglial cell death might influence NPCs in the developing brain. To determine whether there were NPCs in the GFP^+^PI^+^ clusters, we performed our LSFM analysis on *Cx3cr1^GFP/+^* mice, with the addition of anti-Pax6 antibody to stain Pax6^+^ NPCs in the brain. Our imaging showed that the GFP^+^PI^+^ clusters were indeed associated with NPCs in the fetal brain (Fig. 4*A*). Based on this result, we hypothesized that defective lytic cell death of microglia might contribute to alterations in brain structures resulting from improper development of NPCs and neurons derived from the LGE and iTh. To test our hypothesis, we measured the volumes of major parts of the cerebrum as well as descendants of the LGE (striatum) and iTh (thalamus) and found that there were no differences between adult WT and *Casp1*^−/−^ mice (Extended Data Fig. 4-1) in these areas.

10.1523/ENEURO.0342-20.2020.f4-1 Extended Data Figure 4-1 Brain volume does not change in *Casp1*^−/−^ mice. Volumes of the cerebrum, thalamus, and striatum of adult male WT (black circles) and *Casp1*^−/−^ (red triangles) mouse brains were determined by Nissl staining and stereology. Cerebrum *p *=* *0.8580, df = 4, *t *=* *0.191. Thalamus *p *=* *0.5247, df = 4, *t *=* *0.691. Striatum *p *=* *0.9372, df = 4, *t *=* *0.084. Data are shown as volumes (μm^3^) of regions from individual mice and their averages. Error bars indicate SEM; n.s., not significant. Download Figure 4-1, TIF file.

Because we did not observe gross structural anomalies in the thalamus, or striatum, we next tested whether we could observe finer compositional changes. Since previous reports have suggested that IL-1β plays important roles in NPC death, proliferation, and differentiation (Crampton et al., 2012; Guadagno et al., 2015), and because the timing of these events are before circuit establishment, we hypothesized that defective microglial lytic cell death may change the numbers of cells located within structures descending from the LGE and iTh. Therefore, we serially sectioned entire cerebrums of adult WT and *Casp1*^−/−^ mice and then immunostained them with anti-NeuN antibody to identify neurons. While we did not find obvious cellular changes in regions descending from the LGE, we found increased numbers of neurons in the TRN (parvalbumin^+^ cells) a region developed from the iTh, in *Casp1*^−/−^ mice (Fig. 4*B*,*C*). These results suggest that appropriate microglial lytic cell death may be important for regulating proper neuronal numbers in certain brain compartments.

### Increased microglial numbers are not observed in the TRN of adult *Casp1*^−/−^ mice

Since the death of fetal microglia in *Casp1*^−/−^ mice was impaired, and microgliosis (increased numbers of microglia) is often associated with pathologic conditions in the brain (Lull and Block, 2010; Brück et al., 2016), we hypothesized that the number of microglia in the TRN might be increased, resulting in disrupted neuronal homeostasis. To test this hypothesis, we measured the number of microglia using anti-Iba1 antibody staining in the TRN. Our results showed that despite the lack of lytic cell death in *Casp1*^−/−^ mice, microglial numbers in the TRN were not increased (Fig. 4*D*,*E*), suggesting that the behavior abnormalities observed in these mice are not because of microgliosis in the brain.

### Injecting inflammasome pathway inhibitors into dams results in behavioral abnormalities in offspring

Since we observed that lytic cell death occurred on specific days during fetal brain development and microglial death might be necessary for the proper differentiation of NPCs, we hypothesized that inhibition of the NLRP3-CASP1-GSDMD/IL-1β pathway during this developmental window might be sufficient to induce behavior abnormalities similar to those observed in *Casp1*^−/−^ mice. To test this hypothesis, WT pregnant mothers were injected with specific drug inhibitors of CASP1 and NLRP3 (VX-765 and MCC950, respectively; Coll et al., 2015) at E12.5, E13.5, and E14.5, times when we observed lytic cell death in WT fetal brains, and then behavior assays were performed on the offspring to determine their activity and anxiety levels (Fig. 5*A*). Our results showed that male offspring from MCC950-injected mice (and, to a lesser extent, VX-765-injected mice) showed hyperactivity (Fig. 5*B*) and low anxiety levels (Fig. 5*C*; Extended Data Fig. 5-1*A*,*B*). These results suggest that pharmacological disruption of the NLRP3/CASP1 pathway, especially NLRP3 inhibition (MCC950), during development may be sufficient to induce the behavior abnormalities observed in our knock-out mice. Our working model is that the NLRP3-CASP1-GSDMD/IL-1β cascade in microglia is essential for normal brain development, and that genetic or pharmacological disruption of this pathway leads to aberrant behavior in mice (Fig. 5*D*). We also propose that disruption of this pathway may impact the development of NPCs, thus resulting in increased numbers of neurons in the TRN, a region descendant from the iTh, which may cause atypical behaviors in mice.

10.1523/ENEURO.0342-20.2020.f5-1 Extended Data Figure 5-1 Evaluation of inhibitor-exposed offspring in the elevated plus maze and our working model of the role of lytic microglial death in NPC development. ***A***, Elevated plus maze assay results supporting Figure 5*C* are shown as the time spent in open arms compared to the total time spent on the apparatus (%). ***B***, Elevated plus maze assay results supporting Figure 5*C* are shown as the number of entries into open arms compared to the total number of entries (%). ***A***, *p *=* *0.0325, df = 28, *F* = 3.921. Tukey’s multiple comparison test: Veh versus VX-765 *p = *0.1736, Veh versus MCC950, *p *=* *0.0307, VX-765 versus MCC950 *p *=* *0.6151. ***B***, *p *=* *0.1981, df = 28, *F* = 1.724. Tukey’s multiple comparison test: Veh versus VX-765 *p = *0.4081, Veh versus MCC950 *p *=* *0.2001, VX-765 versus MCC950 *p *=* *0.8550; *p <* *0.05; n.s., not significant. ***C***, Cartoon describing our working model of the fetal brain iTh at E14.5. Damage-associated molecular patterns (DAMPs), such as ATP, are released from dead or dying neural cells to activate microglial NLRP3 and initiate the NLRP3-CASP1-GSDMD/IL-1β cascade depicted in Figure 5*D*. As a result, sterile inflammation (perhaps mediated in part by IL-1β) influences the development of NPCs. We propose that proinflammatory cytokines are required to promote the death of TRN precursor cells in the iTh, which is why we observe increased numbers of neurons in the TRN region of adult *Casp1*^−/−^ brains. Download Figure 5-1, TIF file.

Movie 1. Representative LSFM imaging of male E14.5 WT fetal brain. Embryos had one copy of *Cx3cr1-*GFP and were injected with PI 10–30 min before tissue harvest and visualization. Movies show sequential *z*-planes of a typical acquisition followed by 3D maximum intensity projection. Purple, PI; green, *Cx3cr1*-GFP; white, merged. 10.1523/ENEURO.0342-20.2020.video.1

Movie 2. Representative LSFM imaging of male E14.5 *Casp1*^−/−^ fetal brain. Embryos had one copy of *Cx3cr1-*GFP and were injected with PI 10–30 min before tissue harvest and visualization. Movies show sequential *z*-planes of a typical acquisition followed by 3D maximum intensity projection. Purple, PI; green, *Cx3cr1*-GFP; white, merged. 10.1523/ENEURO.0342-20.2020.video.2

## Discussion

In this study, we have shown that the NLRP3-CASP1-GSDMD/IL-1β cascade in microglia is required for normal brain development and prevents behavior abnormalities such as hyperactivity and low anxiety levels (Fig. 1). *Casp1* and *Nlrp3* are predominantly expressed in CX3CR1^+^ myeloid-lineage cells in the fetal brain, and our data showed that the re-expression of *Casp1* in these cells in *Casp1*-deficient mice restored normal behaviors (Fig. 2). These results indicate the importance of myeloid cells, particularly microglia, in early brain development to ensure normal behaviors in mice.

The current study shows that microglial lytic cell death occurs in a spatiotemporal manner, with small subsets of microglia in the iTh and LGE undergoing death from E12.5 to E14.5. The timing of microglial death during development is essential since NPCs start to differentiate at E12.5 in the mouse brain. It is also possible that active cell death occurs in NPCs and newly differentiated neurons in and around this timeframe. Thus, dead or dying NPCs and neurons could release ATP and K^+^ that could trigger the NLRP3 cascade in microglia, resulting in the cleavage of pro-IL1β to its mature form (He et al., 2016). It has been reported that microglial IL-1β is required for NPC differentiation and death (Crampton et al., 2012; Guadagno et al., 2015), so we attempted to measure the amount of secreted IL-1β in the ventricles of the fetal brain by ELISA assay. IL-1β levels were found to be below the level of detection (data not shown), but we assume that this was because only a small number of cells underwent lytic cell death (Extended Data Fig. 3-1*B*). However, despite the undetectable levels of IL-1β in the fetal brain, we and another group found that *Il-1r*^−/−^ mice exhibit behavior abnormalities (Murray et al., 2013 and this article), suggesting that IL-1β does indeed play a role in ensuring normal behavior development in mice.

Since we observed that Pax6^+^ NPCs were a part of PI^+^*Cx3cr1*-GFP^+^ clusters (Fig. 4*A*), and neural circuits are important for behavior, we reasoned that we should look for alterations in neurons as a consequence of defective lytic cell death in *Casp1*^−/−^ microglia to explain the atypical behaviors observed in our inflammasome pathway knock-out mice. We did not observe global changes in brain structures in the striatum and thalamus descendent from the LGE and iTh (Extended Data Fig. 4-1) in adult *Casp1*^−/−^ brains; however, we did confirm increased numbers of neurons in the TRN (Fig. 4*B*,*C*), a region descending from the microglial lytic cell death zone observed in developing *Casp1*^−/−^ mice. Interestingly, a recent report suggested that the TRN regulates a circuit required for attention and hyperactivity (Wells et al., 2016), behavior abnormalities that were observed in *Casp1*^−/−^ mice (Fig. 1*A*,*B*; Extended Data Fig. 1-1*B–D*). As discussed above, this increase in neurons could have been because of a lack of IL-1β production in microglia that resulted in impaired death and/or differentiation of NPCs. Alternatively, we could speculate that microglial engulfment of NPCs might be decreased since we observed that mRNA expression of *Mrc2*, a gene known to be involved in phagocytosis, was decreased in *Casp1*^−/−^ microglia isolated from adult mice (data not shown). However, since we did not observe the deregulation of other genes regulating phagocytosis in *Casp1*^−/−^ microglia isolated from adult mice, it is difficult to conclude whether this process is actually impaired. Thus, it would be of interest to study the phagocytic activity of microglia in mice deficient for genes regulating the NLRP3-CASP1-GSDMD/IL-1β cascade. Furthermore, additional studies are required to determine whether the functions of neurons in the TRN are also altered to explain the behavior abnormalities observed in mice.

Since increased numbers of microglia (microgliosis) are often associated with pathologic conditions, such as neurodegenerative diseases (Lull and Block, 2010; Brück et al., 2016), we expected that defective microglial death would lead to increased microglial cell numbers in regions descendent from the iTh and LGE, thus altering neural functions. However, we did not observe increased numbers of microglia in the TRN. Lytic cell death of microglia was found to occur primarily in the LGE and iTh regions of the brain. Recent reports suggest that microglia might be transcriptionally heterogenous, indicating that specific microglial subsets may exist in different areas in the brain (Li et al., 2019; Masuda et al., 2019). If this is the case, the lytic cell death that we observed may be occurring only in specific subsets of microglia located in these regions. Based on these results, it is clear that specific microglial lytic cell death has an impact on normal brain development; however, the precise mechanism of this regulation and the interplay between specific microglia and NPCs/neurons in the developing brain warrant further study.

Impressively, injection of NLRP3 and CASP1 inhibitors into pregnant WT mice during the time frame of microglial death resulted in behavior abnormalities in their offspring that recapitulated those observed in the NLRP3 pathway knock-out mice. These results suggest that disruption of this pathway during this key developmental window is sufficient to significantly alter normal brain development and behavior. Since the NLRP3-CASP1-GSDMD/IL-1β cascade in microglia is crucial for normal brain development, and either genetic or pharmacological disruption of this pathway can lead to atypical behavior in mice (Fig. 5*D*), we propose that pro-inflammatory cytokines, such as IL-1β, may play essential roles in regulating the differentiation and proliferation of NPCs (Extended Data Fig. 5-1*C*). Therefore, the numbers and functions of neurons descending from the LGE and iTh may be altered in mice with defects in the NLRP3-CASP1-GSDMD/IL-1β cascade, which could contribute to their behavioral phenotypes.

## Synthesis

Reviewing Editor: Aniko Korosi, University of Amsterdam Decisions are customarily a result of the Reviewing Editor and the peer reviewers coming together and discussing their recommendations until a consensus is reached. When revisions are invited, a fact-based synthesis statement explaining their decision and outlining what is needed to prepare a revision will be listed below. The following reviewer(s) agreed to reveal their identity: Yvonne Nolan.

Reviewer 1

This manuscript presents a timely and important investigation of the role of the inflammasome cascade in microglia in fetal brain development and later behavior in adulthood. The authors should be commended for their behavioral analysis of a large number of different genotypes in order to support their conclusion that the inflammasome cascade is important for normal behavior. In addition, they have performed a time-limited pharmacological manipulation in order to demonstrate the importance of the inflammasome specifically during E12.5-E14.5. Finally, they have also completed a critical “rescue” experiment, which is rare except in the highest-tier journals, where they have induced Casp1 expression in just CX3CR1+ cells, and rescued the atypical behavior. However, there are some statistical errors and omission of methodological details that are required for reproducibility, as well as some missing analyses (actual counts of microglial numbers) that reduce my enthusiasm for the manuscript. If these errors can be corrected and the proper analyses added, then I believe the manuscript would be a good fit for eNeuro. Please see below for a list of my specific comments in the order that they appear in the manuscript:

• Methods, page 4, last paragraph: You say that your wild-type mice were purchased from Charles River and Jax, but these are actually two different sub-strains of mice, C57BL6/N and C57BL6/J, respectively, which have been shown to have slightly different phenotypes. Please specify exactly which mice were used for which experiments, as a critical factor in reproducibility and transparency.

• Methods, throughout: For each experiment, you should list the total n for each group broken down into the number of males and females in each group, as well as the number of litters that are represented (especially important for the prenatal/fetal experiments).

• Methods, page 5, last two paragraphs: I am more familiar with standard open field assays that are 10 min. and elevated plus maze assays that are 5 min. in length. Please specify why you used longer durations and/or cite references for these durations. Can you also present data (can be in the extended data figures) for the first 10 min. of the open field test and the first 5 min. of the EPM so that readers can more easily compare with other tests performed for the standard durations?

• Methods, page 10, 1st paragraph: You cannot use t-tests for analyses where more than 2 groups are being compared, namely Figure 2B and 2C, and Figure 5B and 5C (as well as Figure 2-1E in the Extended Data). You need to use a One-way ANOVA followed by post-hoc tests that are corrected for multiple comparisons in these instances. You also talk about using Fisher’s exact test to “correlate the appearance of clusters and their correlations with time point and genotype", which is a confusing description. You are not performing correlation analysis, but rather analyzing contingency tables to determine the probability that the distributions are not due to chance.

• Results, page 11, 1st paragraph: “Interestingly, these behavior changes were only observed in male, but not female, Casp1-/- mice (data not shown)”. This is interesting, but it’s then unclear whether the rest of your data is representing both males and females, or whether you have continued to leave out females. Addressing my point above where I ask that you always break down the n by males and females for each experiment and figure will help solve this problem.

• Results, page 16, 2nd paragraph, Fig. 3E: The mean fluorescence intensity of Iba1 does not correspond well to microglial cell numbers, because changes could be due to cell size (or “activation”) rather than number. You should count microglial cells like you did for neurons in order to make any conclusions about microglial numbers.

• Discussion: The discussion feels a bit too short overall. A final summary statement of all of your findings and their implications should be added at the end, rather than ending with a reference to a figure. It also seems like you are missing any discussion of the seemingly contradictory idea that fewer microglia dying during development in Casp1-/- mice results in more neurons in the TRN in adulthood. Many people have previously shown that microglia engulf/phagocytose NPCs and neurons, so shouldn’t more microglia surviving result in fewer neurons? You should explicitly address this idea (citing the relevant literature), as well as presenting your alternative view that microglial-secreted molecules, such as IL-1beta, may change neuronal number independently of phagocytosis. Of course, you haven’t conclusively shown any change in microglial numbers (you need to count microglia as mentioned in the point above). But beyond counting microglia in adulthood, it would also be useful to count them during gestation, not just in the clusters like Fig. 3-1B, but in the whole region of LGE and iTh. For example, does the formation of clusters go along with increased total microglial numbers at this age, or is it just a difference in microglial patterning in these regions? And if microglial are not dying by a lytic cell death, is there an alternative method of cell death (e.g., apoptosis) that is occurring in order to keep microglial cell numbers overall the same? (if you indeed end up finding no difference in number after counting).

• Figure 3A: Some of the text in the labels is obscured by the image of the pre-cleared tissue. Please move it out of the way, in line with the post-cleared tissue image.

• Figure 1-1B: Why is the attention data not in the main figure? Did you only do perform this test for the Casp1-/- mice, or are you just not showing the data from the other genotypes? It should be clearly specified in the methods which genotypes the test was performed for if only a subset, since currently it sounds like you performed all behavior tests for all genotypes.

• Table 1: climbing/3-chamber data- where is this? Don’t remember you mentioning it. I would recommend taking it out of the table if you are not presenting the data.

Reviewer 2

The authors of manuscript entitled “Lytic cell death in specific microglial subsets is required for preventing atypical behaviour in mice” have presented a well-written and succinct manuscript with an array of data that contributes to the understanding of the role of microglia during development in shaping future behaviour. Here are some suggestions for minor improvement to the manuscript:

The introduction sets the context and links clinical conditions to the rationale for the experimental work outlined here. It would benefit from some additional information regarding the specificity of inflammasomes to microglia, and the relevance of Cx3cr1 in the context of the inflammasome, to provide clarity.

For all results, the t value, associated p value and degrees of freedom should be reported.

Given that the various mouse lines in the current series of experiments are hyperactive, it is possible that this hyperactivity could impact that time spent in the open arms of the elevated plus maze. The open-arm time/total time and the number of open arm entries/total entries should be provided.

Distance travelled in the Open Field should be provided in cm rather than inches to align with international practise.

It is interesting that there were increased numbers of neurons in the TRN in Casp1-/- mice. Could the authors hypothesise on the impact that defective microglial lytic cell death may have on the development of astrocytes, as these cell phenotypes are of the same neural lineage (from NPCs) as neurons?

Given that the diameter of GFP+PI+ cells had larger diameters as a measure of lytic cell death, is it possible that phagocytic activity of microglial cells could contribute to their enlargement? A Discussion point on this is warranted.

The authors should provide a graph accompanied by statistical evidence to show that the formation of GFP+PI+ cell clusters in the LGE and iTH was significantly reduced in Cx3cr1-GFP mice at E14.5.

What background strain were the transgenic mice bred on? Was it the same as the wild type control mice?

Table 1 outlines that a three-chamber interaction assay was performed yet no detail in the methods or results is provided.

For immunofluorescence staining, the thickness of the tissue that was cryostat sectioned should be included.

There are some discrepancies in the labelling of the figures and associated description in the text. For example, there is no Figure 1-1C yet it is referenced in the text of the Results section and Figure legend. There is also a discrepancy with Figure 1-1B. Check out Figure legend 4 too.

On page 13, a reference to support the statement that “Gsdmd is essential for the induction of lytic cell death” is needed.

On page 18, a reference to support the statement that “we and another group found that Il-1r-/- exhibit behaviour abnormalities” should be provided.

Were all experiments performed in males? It’s not quite clear from the description.

On Figure 5D, it could be useful to portray how the authors propose that neurons fit into the pathways.

The graph of Figure 2-1E should be labelled to indicate which gene is measured.
