# Supplementary figures and images for: Lytic Cell Death in Specific Microglial Subsets Is Required for Preventing Atypical Behavior in Mice
Source: eNeuro. 2021 Jan 12;8(1):ENEURO.0342-20.2020. doi: 10.1523/ENEURO.0342-20.2020 (PMC7877467; doi:10.1523/ENEURO.0342-20.2020)

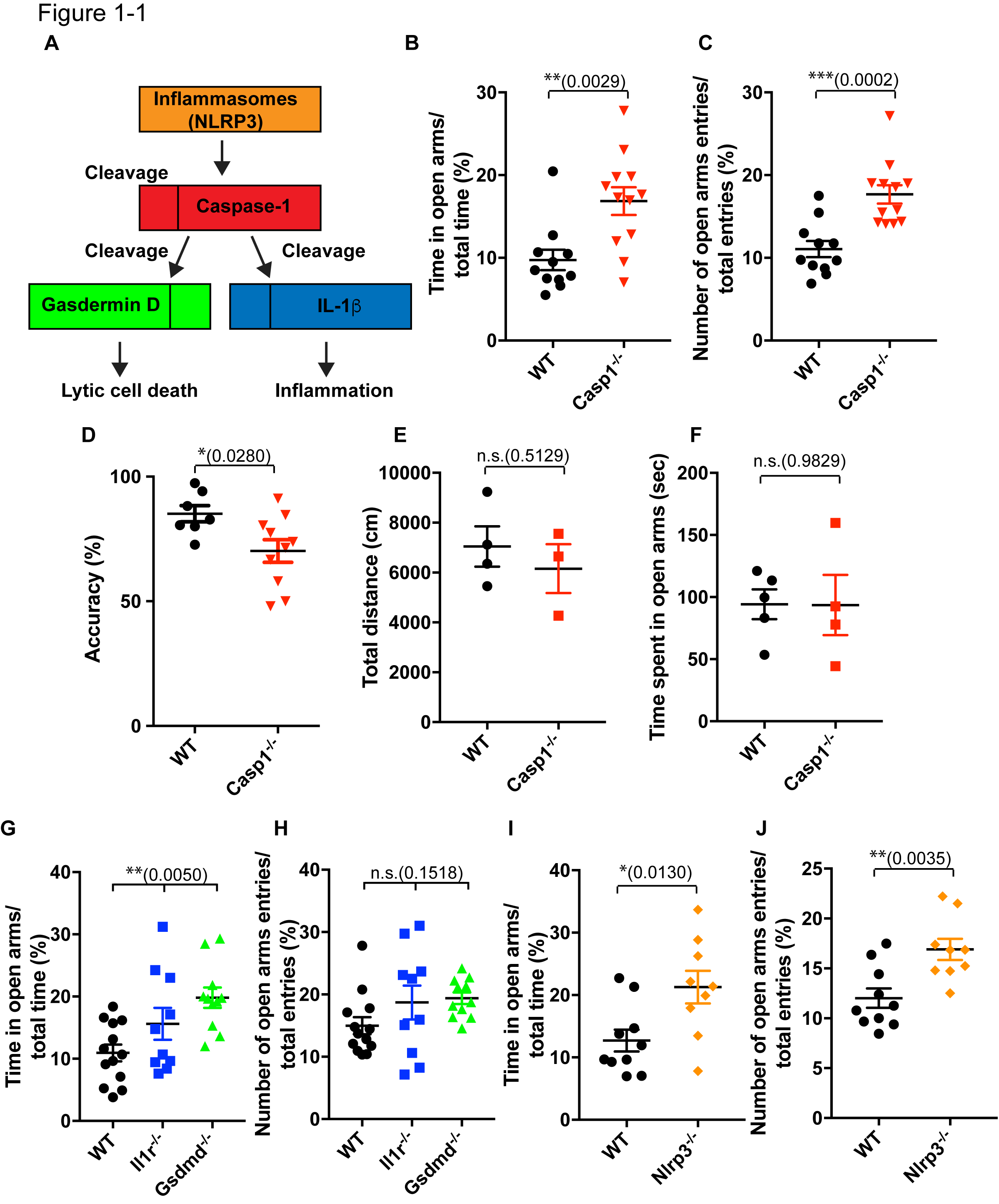

Supplement: Extended Data Figure 1-1 — Inflammasome cascade and attention behavior of Casp1−/− mice. A, Diagram of the inflammasome cascade. NLRP3, an inflammasome protein, is activated and provides a platform to cleave pro-CASP1 to generate CASP1. CASP1 cleaves pro-GSDMD to GSDMD, which assembles and generates pores in the plasma membrane and induces lytic cell death. CASP1 also cleaves pro-IL-1β to generate mature IL-1β cytokine. B, G, I, Elevated plus maze assay results from the indicated male mice to support Figure 1B,D,F are shown as the time spent in open arms compared to the total time spent on the apparatus (%). C, H, J, Elevated plus maze assay results from the indicated male mice to support Figure 1B,D,F are shown as the number of entries into open arms compared to the total number of entries (%). D, Attention behavior of male WT (N = 7, black circles, from 3 litters) and Casp1−/− (N = 10, red triangles, from 4 litters) mice was determined by the 5-CSRTT and is shown as the accuracy of their responses (%). E, General activity of female WT (black circles, N = 4, from 2 litters) and Casp1−/− (red squares, N = 3, from 2 litters) mice was determined by open field assay and is shown as total distance moved (centimeters). F, Anxiety levels of female WT (black circles, N = 5) and Casp1−/− (red squares, N = 4) mice were determined using the elevated plus maze assay and are shown as the time spent in open arms (seconds). Dots indicate individual animals and error bars show SEM. B, p = 0.0029, df = 21, t = 3.371. C, p = 0.0002, df = 21, t = 4.425. D, p = 0.028, df = 15, t = 2.432. E, p = 0.5129, df = 5, t = 0.704. F, p = 0.9829, df = 7, t = 0.022. G, p = 0.0050, df = 33, F = 6.304. Tukey’s multiple comparison test: WT versus Il-1r−/− p = 0.1809, WT versus Gsdmd−/− p = 0.0036, Il-1r−/− versus Gsdmd−/− p = 0.2729. H, p = 0.1518, df = 33, F = 2.005. Tukey’s multiple comparison test: WT versus Il-1r−/− p = 0.2964, WT versus Gsdmd−/− p = 0.1717, Il-1r−/− versus Gsdmd−/− p = 0.9610. I, p = 0.0130, df = 17, t = [file enu-eN-NWR-0342-20-s05.tif]

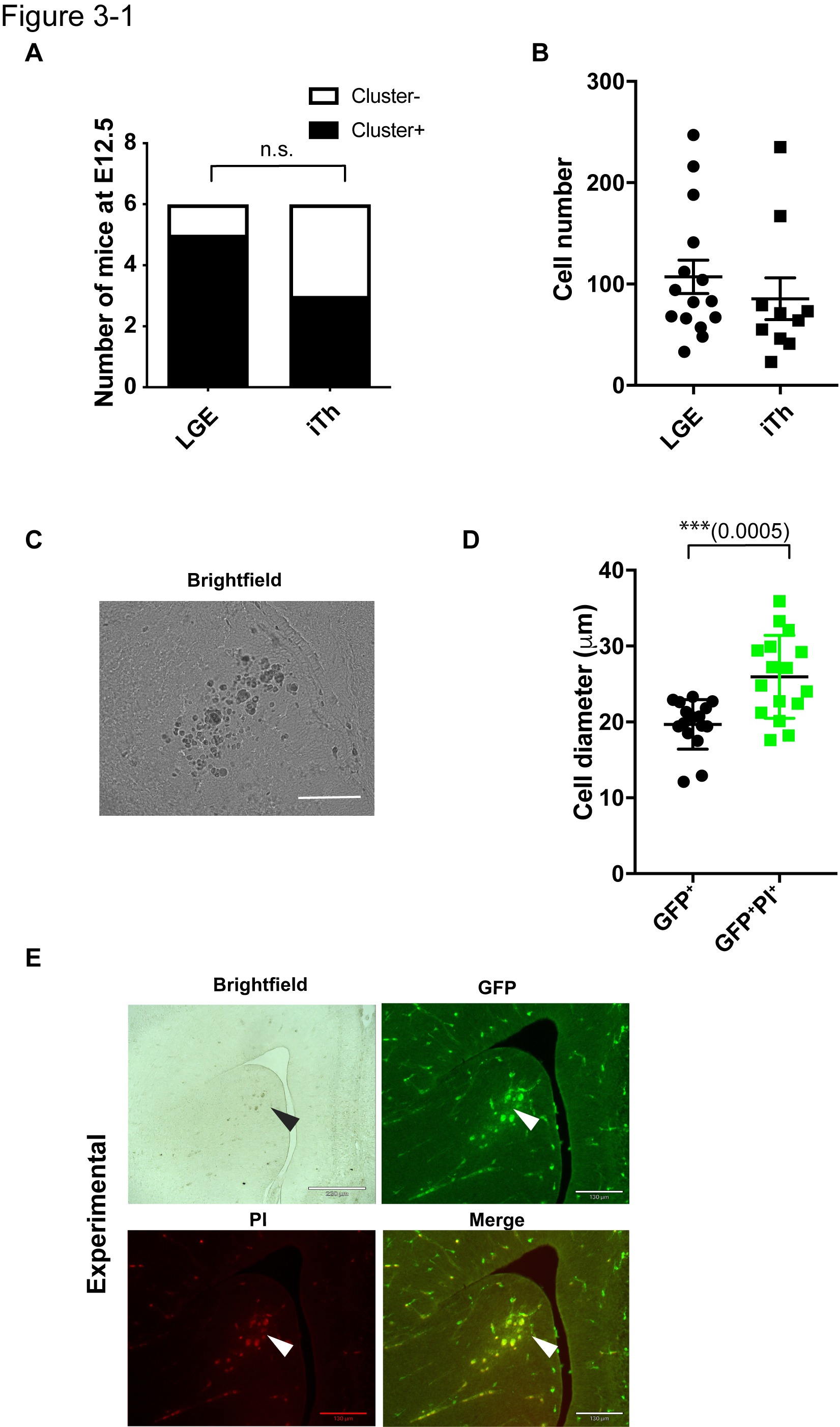

Supplement: Extended Data Figure 3-1 — Quantification and qualification of clusters in WT and Casp1 re-expression mice. A, Numbers of WT mice (male, N = 6) that exhibited clusters in the LGE and iTh at E12.5 are shown; n.s., not significant. B, Cell numbers in the clusters from the LGE and iTh at E14.5 are shown. C, A representative brightfield image of the cluster including swollen microglial cells. Scale bar: 100 μm. D, Diameters of GFP+ and GFP+PI+ cells in the clusters are shown; p = 0.0005, df = 30, t = 3.935. Diameters of individual cells and the averages are shown. Error bars indicate SEM; ***p < 0.001. E, Casp1−/−; iCasp1; Cx3cr1-Cre mice express GFP upon Cre-mediated recombination. Mice were injected with PI as shown in Figure 3A. Representative brightfield (top left), Cx3cr1-GFP staining signal (top right), PI signal (bottom left), and merged (bottom right) images are shown. Scale bar in the bright field image indicates 230 μm and the others indicate 130 μm. Download Figure 3-1, TIF file. [file enu-eN-NWR-0342-20-s03.tif]

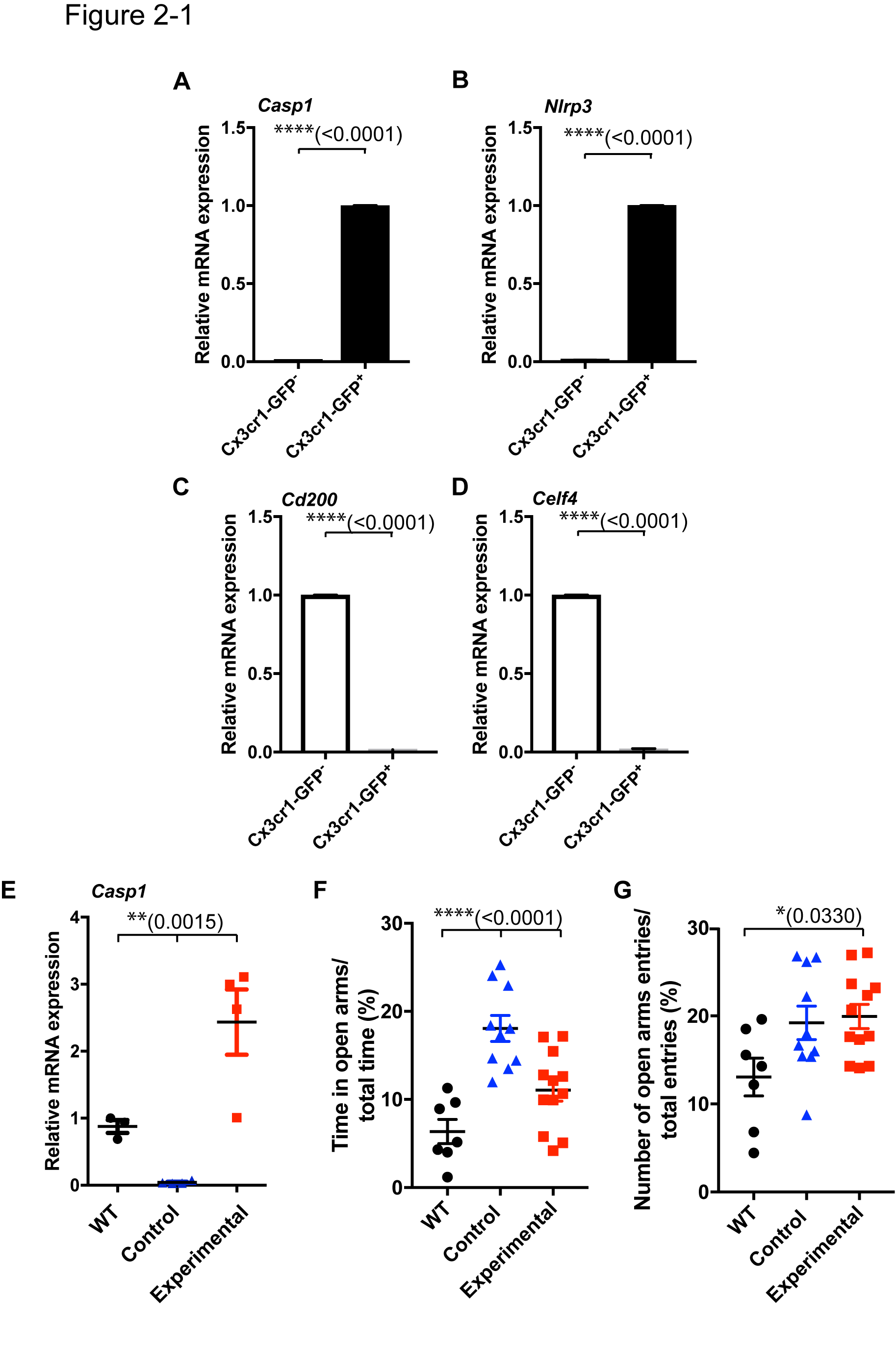

Supplement: Extended Data Figure 2-1 — Casp1 is expressed in microglia from fetal brains and adult Casp1−/−; iCasp1; Cx3cr1-Cre mice. A–D, mRNA expressions of Casp1 (A), Nlrp3 (B), Cd200 (C), and Celf4 (D) in Cx3cr1-GFP+ and Cx3cr1-GFP- cells from male fetal brain at E14.5, as determined by RT-qPCR. A, p < 0.0001, df = 2, t = 688.100. B, p < 0.0001, df = 2, t = 1319.000. C, p < 0.0001, df = 2, t = 598.900. D, p < 0.0001, df = 2, t = 220.900. Data are shown as the average of duplicates, and error bars indicate SEM. E, Microglial cells were isolated from the brains of adult male WT (black circles), control (blue rectangles), and experimental (red rectangles) mice and the mRNA expressions of Casp1 were determined by RT-qPCR. F, Elevated plus maze assay results supporting Figure 2C are shown as the time spent in open arms compared to the total time spent on the apparatus (%). G, Elevated plus maze assay results supporting Figure 2C are shown as the number of entries into open arms compared to the total number of entries (%). E, p = 0.0015, df = 10, F = 16.19. Tukey’s multiple comparison test: WT versus control p = 0.2260, WT versus experimental p = 0.0230, control versus experimental p = 0.0013. F, p < 0.0001, df = 28, F = 15.92. Tukey’s multiple comparison test: WT versus control p < 0.0001, WT versus experimental p = 0.0759, control versus experimental p = 0.0024. G, p = 0.0330, df = 28, F = 3.902. Tukey’s multiple comparison test: WT versus control p = 0.0743, WT versus experimental p = 0.0340, control versus experimental p = 0.9469. Data indicates individual mice and averages are shown. Error bars indicate SEM; **p < 0.01, ****p < 0.0001. Download Figure 2-1, TIF file. [file enu-eN-NWR-0342-20-s04.tif]

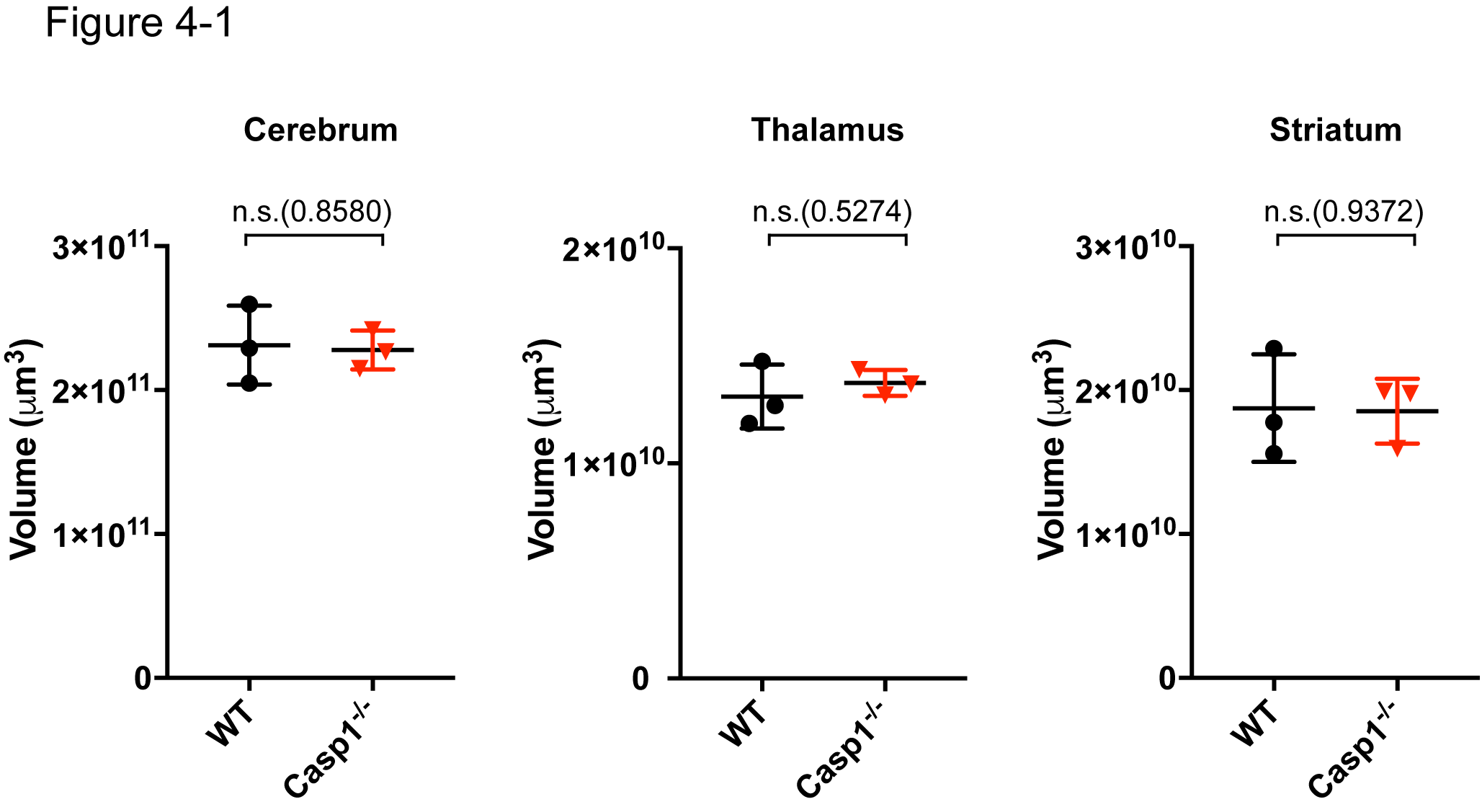

Supplement: Extended Data Figure 4-1 — Brain volume does not change in Casp1−/− mice. Volumes of the cerebrum, thalamus, and striatum of adult male WT (black circles) and Casp1−/− (red triangles) mouse brains were determined by Nissl staining and stereology. Cerebrum p = 0.8580, df = 4, t = 0.191. Thalamus p = 0.5247, df = 4, t = 0.691. Striatum p = 0.9372, df = 4, t = 0.084. Data are shown as volumes (μm3) of regions from individual mice and their averages. Error bars indicate SEM; n.s., not significant. Download Figure 4-1, TIF file. [file enu-eN-NWR-0342-20-s02.tif]

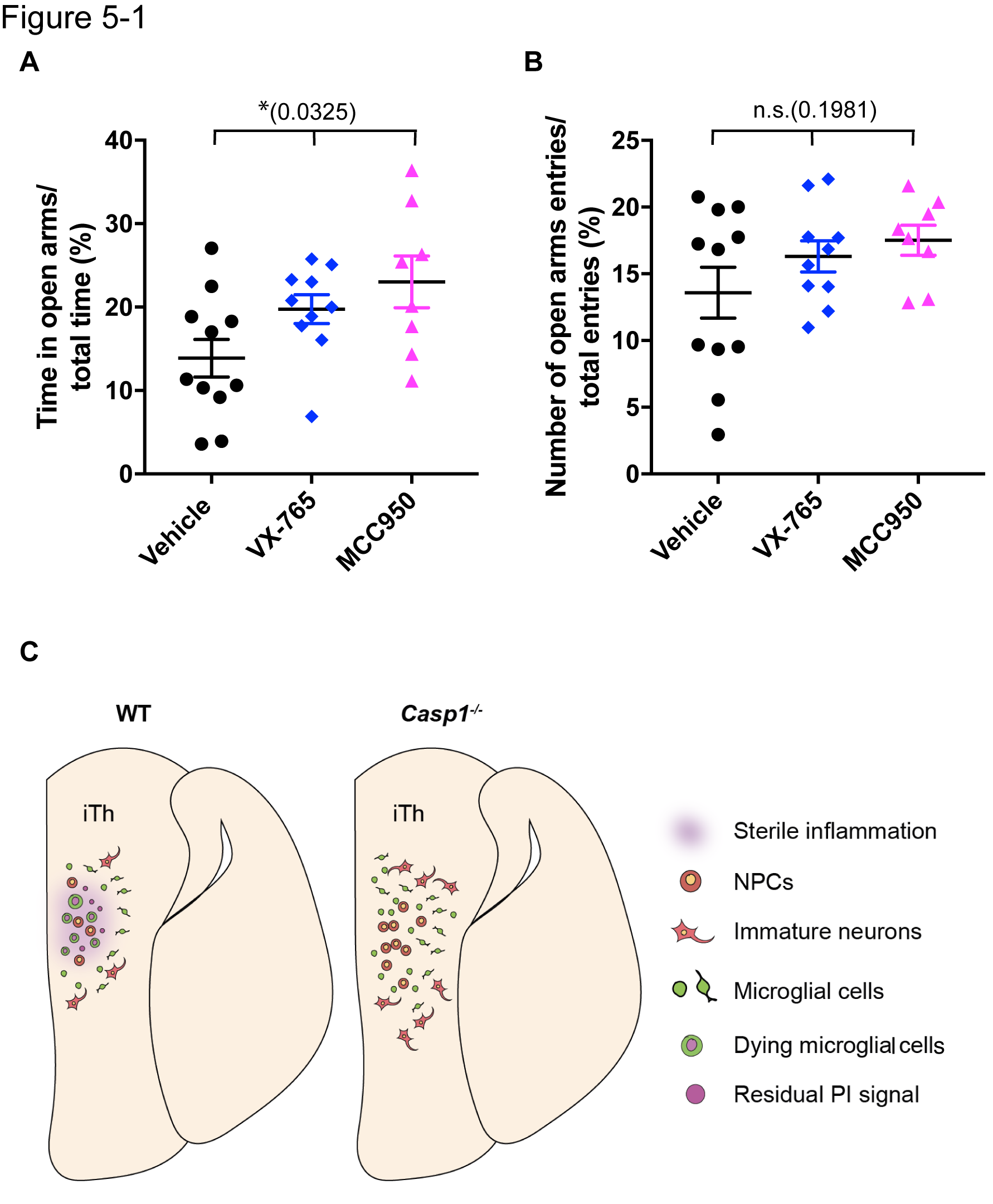

Supplement: Extended Data Figure 5-1 — Evaluation of inhibitor-exposed offspring in the elevated plus maze and our working model of the role of lytic microglial death in NPC development. A, Elevated plus maze assay results supporting Figure 5C are shown as the time spent in open arms compared to the total time spent on the apparatus (%). B, Elevated plus maze assay results supporting Figure 5C are shown as the number of entries into open arms compared to the total number of entries (%). A, p = 0.0325, df = 28, F = 3.921. Tukey’s multiple comparison test: Veh versus VX-765 p = 0.1736, Veh versus MCC950, p = 0.0307, VX-765 versus MCC950 p = 0.6151. B, p = 0.1981, df = 28, F = 1.724. Tukey’s multiple comparison test: Veh versus VX-765 p = 0.4081, Veh versus MCC950 p = 0.2001, VX-765 versus MCC950 p = 0.8550; *p < 0.05; n.s., not significant. C, Cartoon describing our working model of the fetal brain iTh at E14.5. Damage-associated molecular patterns (DAMPs), such as ATP, are released from dead or dying neural cells to activate microglial NLRP3 and initiate the NLRP3-CASP1-GSDMD/IL-1β cascade depicted in Figure 5D. As a result, sterile inflammation (perhaps mediated in part by IL-1β) influences the development of NPCs. We propose that proinflammatory cytokines are required to promote the death of TRN precursor cells in the iTh, which is why we observe increased numbers of neurons in the TRN region of adult Casp1−/− brains. Download Figure 5-1, TIF file. [file enu-eN-NWR-0342-20-s01.tif]
